# The Fra-1–miR-134–SDS22 feedback loop amplifies ERK/JNK signaling and reduces chemosensitivity in ovarian cancer cells

**DOI:** 10.1038/cddis.2016.289

**Published:** 2016-09-29

**Authors:** Jianmin Wu, Yimin Sun, Pei-Ying Zhang, Mengyao Qian, Hengchao Zhang, Xiao Chen, Di Ma, Yunsheng Xu, Xiaoming Chen, Kai-Fu Tang

**Affiliations:** 1 Institute of Genomic Medicine, Wenzhou Medical University, Wenzhou 325000, Zhejiang, China; 2 Institute of Translational Medicine, First Affiliated Hospital of Wenzhou Medical University, Wenzhou 325015, Zhejiang, China; 3 National Engineering Research Center for Beijing Biochip Technology, Beijing 102206, China; 4 Digestive Cancer Center, First Affiliated Hospital of Wenzhou Medical University, Wenzhou 325015, Zhejiang, China; 5 Department of Dermato-Venereology, First Affiliated Hospital of Wenzhou Medical University, Wenzhou 325015, Zhejiang, China; 6 Department of Pediatric Surgery, First Affiliated Hospital of Wenzhou Medical University, Wenzhou 325015, Zhejiang, China

## Abstract

The Fra-1 transcription factor is frequently upregulated in multiple types of tumors. Here we found that Fra-1 promotes miR-134 expression. miR-134 activates JNK and ERK by targeting SDS22, which in turn induces Fra-1 expression and leads to miR-134 upregulation. In addition, miR-134 augmented H2AX S139 phosphorylation by activating JNK and promoted non-homologous end joining (NHEJ)-mediated DNA repair. Therefore, ectopic miR-134 expression reduced chemosensitivity in ovarian cancer cells. Furthermore, miR-134 promotes cell proliferation, migration and invasion of ovarian cancer cells, and enhances tumor growth *in vivo*. Of particular significance, both Fra-1 and miR-134 are upregulated in ovarian cancer tissues, and Fra-1 and miR-134 expression is positively correlated. High levels of miR-134 expression were associated with a reduced median survival of ovarian cancer patients. Our study revealed that a Fra-1-miR-134 axis drives a positive feedback loop that amplifies ERK/JNK signaling and reduces chemosensitivity in ovarian cancer cells.

MicroRNAs (miRNAs) are non-coding RNAs 21–23 nucleotides in length that repress the expression of target messenger RNAs (mRNAs) and participate in multiple steps during cellular transformation and tumorigenesis.^1, 2^ miR-134 was initially characterized as a brain-specific miRNA that regulates synaptic development, synaptic plasticity and memory formation.^3, 4^ Silencing miR-134 in experimental models of status epilepticus suppresses prolonged seizures and exerts neuroprotective effects.^5^ miR-134 also regulates the proliferation and differentiation of embryonic stem cells and cancer stem cells. miR-134 enhances the differentiation of mouse embryonic stem cells by inhibiting Nanog and LRH1 expression^6^ and suppresses proliferation and migration in cancer stem cells.^7, 8^ Recent evidence has revealed that miR-134 also has pivotal roles in tumor transformation and progression. It exhibits oncogenic activity in head and neck carcinoma and lung adenocarcinoma^9, 10, 11^ as well as tumor suppressive activity in breast cancer, glioma, hepatocellular carcinoma, osteosarcoma and renal cell carcinoma.^8, 12, 13, 14, 15^

Ras, a 21-kDa small GTPase that regulates cell proliferation and differentiation, has a critical role in cancer initiation and progression. Mutations in Ras family genes (H-Ras, K-Ras and N-Ras) have been detected in ~30% of human cancers.^16, 17^ Ras activation leads to the membrane recruitment and activation of RAF proteins, which subsequently leads to the activation of mitogen-activated protein kinase kinase (MEK). Activated MEK phosphorylates mitogen-activated protein kinase (MAPK), which can directly and indirectly activate multiple transcription factors, including activator protein-1 (AP-1).^18, 19^ The AP-1 complex is a dimer composed of transcription factors from the Jun (c-Jun, JunB and JunD) and Fos (c-Fos, FosB, Fra-1 and Fra-2) families.^20, 21^ Fra-1 is frequently upregulated in a wide variety of tumors, and Fra-1 upregulation is essential for oncogenic H-Ras-induced transformation.^22, 23^ Fra-1 has important roles during consecutive stages of multistep tumor progression by promoting cell proliferation, inhibiting apoptosis and enhancing tumor angiogenesis.^24, 25, 26^ In addition, Fra-1 promotes tumor heterogeneity.^24^

In the current study, we demonstrated that Fra-1 induces miR-134 expression in ovarian cancer cells. Moreover, we identified a miR-134-mediated positive feedback loop that amplifies ERK and JNK signaling, and reduces chemosensitivity in ovarian cancer cells.

## Results

### Fra-1 upregulation induced by oncogenic H-Ras promotes miR-134 expression in ovarian cancer cells

**Figure 1:**
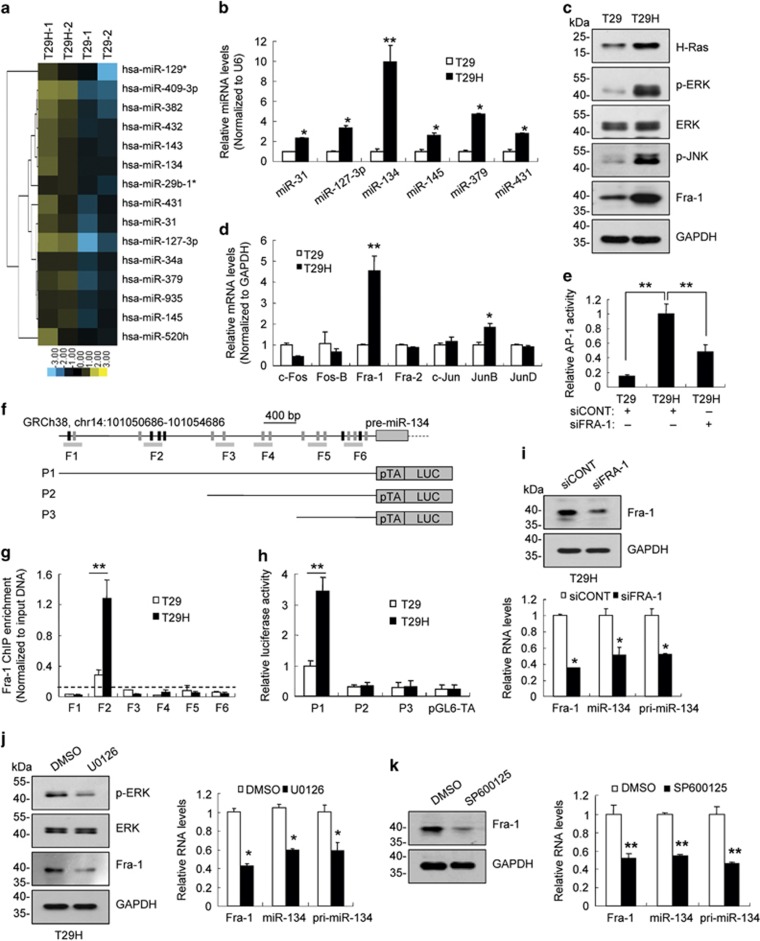
Oncogenic Ras induced miR-134 expression via the ERK/JNK-AP-1 signaling pathways. (**a**) Heat map representation of the top 15 upregulated miRNAs in T29H cells compared with T29 cells. Rows, miRNAs; columns, profiled samples. (**b**) Quantification of six miRNAs in T29 and T29H cells using real-time RT-PCR. (**c**) Representative western blot images of H-Ras, p-ERK, ERK, p-JNK and Fra-1. GAPDH was used as loading control. (**d**) Quantification of AP-1 subunit levels in T29 and T29H cells using real-time RT-PCR. (**e**) Fra-1 knockdown inhibits AP-1 activity in T29H cells. (**f**) Diagram of the 4-kb region upstream of pre-miR-134. The AP-1 (gray and black box) and Fra-1 (black box) binding sites, the amplicons (F1–F6) utilized for ChIP PCR, and the DNA fragments incorporated into luciferase reporter constructs (P1–P3) were indicated. (**g**) ChIP assays with anti-Fra-1 in T29H and T29 cells. (**h**) Luciferase reporter assays with the various reporter constructs in T29 and T29H cells. (**i**) Fra-1 knockdown reduces miR-134 and pri-miR-134 levels in T29H cells. Fra-1 protein levels (top) and the relative RNA expression levels of Fra-1, miR-134 and pri-miR-134 (bottom). (**j**) The effect of MEK1/2 inhibitor U0126 on the miR-134 and pri-miR-134 abundance in T29H cells. The protein levels of ERK, p-ERK and Fra-1 (left) and the relative RNA levels of Fra-1, miR-134 and pri-miR-134 (right). (**k**) The protein levels of Fra-1 (left) and the relative RNA expression levels of Fra-1, miR-134 and pri-miR-134 (right) in T29H cells treated with the JNK inhibitor SP600125. Data are shown as mean±S.D. from three independent experiments. *P<0.05, **P<0.01 by Student's *t*-test

To identify Ras-regulated miRNAs, we profiled the expression of 847 miRNAs using a microarray assay in two human ovarian epithelial cell lines. One cell line was immortalized with SV40 T/t antigens and the human catalytic subunit of telomerase (T29), and the second cell line was transformed with an additional oncogenic H-Ras^V12^ allele (T29H).^27, 28^ A total of 309 miRNAs were differentially expressed in T29 compared with T29H cells (Figure 1a; Supplementary Table S1). The upregulation of six miRNAs (including miR-31, miR-127-3p, miR-134, miR-145, miR-379 and miR-431) was further validated using real-time reverse transcription-polymerase chain reaction (RT-PCR; Figure 1b). miR-134, the most upregulated miRNA, was selected for further in-depth analysis. Transiently expression of H-Ras^V12^ promoted miR-134 expression (Supplementary Figure S1a), and inhibiting Ras signaling using H-Ras^V12^ knockdown or by treating cells with the Ras inhibitor FTI-277 suppressed miR-134 expression (Supplementary Figures S1b and c). These results indicate that activation of Ras signaling promotes miR-134 expression.

Ras activation leads to MAPK-mediated upregulation and phosphorylation of AP-1 subunits and enhances AP-1 activity, thereby inducing downstream gene expression.^18, 19, 29, 30, 31^ We found that the levels of phosphorylated extracellular signal-regulated kinase (p-ERK) and phosphorylated c-Jun NH2 kinase (p-JNK) were increased in T29H cells compared with T29 cells (Figure 1c). In addition, the levels of Fra-1 were ~5-fold increased in T29H cells compared with T29 cells (Figures 1c and d). Consistently, AP-1 activity was greater in T29H cells compared with T29 cells, and Fra-1 knockdown reduced AP-1 activity (Figure 1e). Using bioinformatics analysis, we identified 18 AP-1 binding sites (including 6 Fra-1 binding sites) in a genomic region 4 kb upstream of miR-134 (Figure 1f). Chromatin immunoprecipitation (ChIP) experiments revealed that Fra-1 binding was enriched in a region ~3 kb upstream of miR-134, and that this enrichment was increased in T29H cells compared with T29 cells (Figures 1f and g; Supplementary Figure S2). A luciferase reporter assay revealed that the 4 kb sequence enhanced the activity of a minimal TA promoter (Figures 1f and h), and this enhancer activity was greater in T29H cells compared with T29 cells (Figure 1h). Fra-1 knockdown significantly reduced pri-miR-134 and miR-134 levels (Figure 1i). Moreover, treatment with the MEK1/2 inhibitor U0126 reduced ERK phosphorylation and Fra-1 expression, thereby inhibiting pri-miR-134 and miR-134 expression (Figure 1j). Furthermore, treatment with the JNK inhibitor SP600125 also suppressed Fra-1 and miR-134 expression (Figure 1k). Together, these observations indicate that activation of the Ras/MAPK signaling pathway induced miR-134 expression by upregulating Fra-1.

### miR-134 inhibited the expression of SDS22 by binding to its 3′-untranslated region

**Figure 2:**
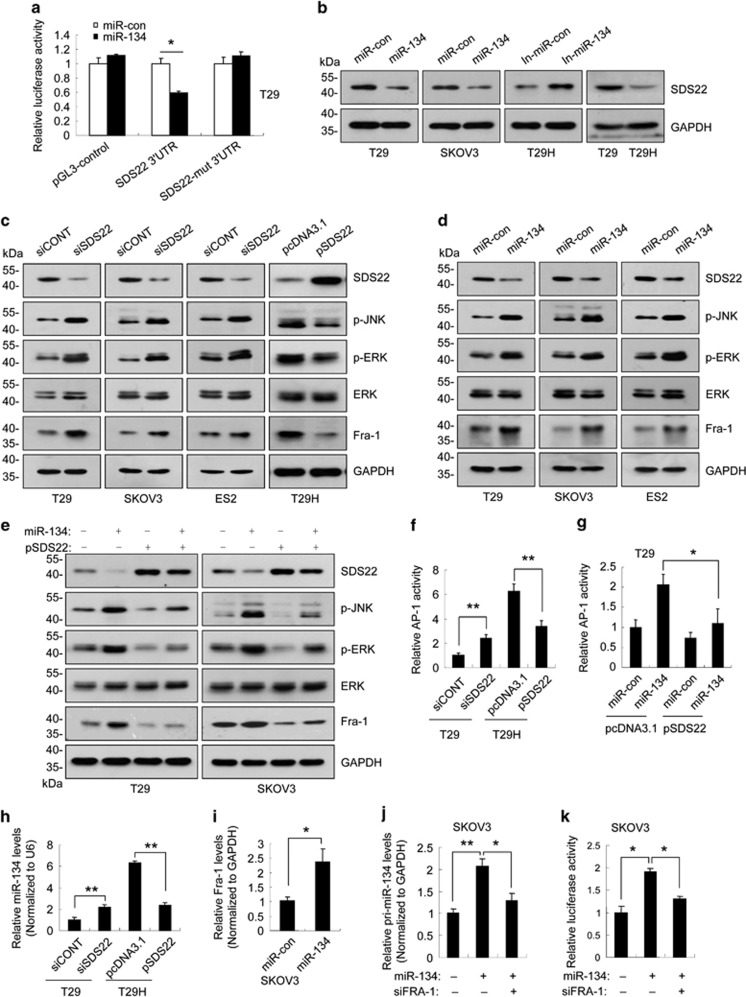
miR-134 drives a positive feedback loop that amplifies ERK/JNK-AP-1 signaling by targeting SDS22. (**a**) The luciferase activity of Luc-SDS22 3′-UTR was inhibited in T29 cells transfected with miR-134 mimics (miR-134). (**b**) SDS22 protein levels in cells transfected with miR-134 mimics or the miR-134 inhibitors (In-miR-134). (**c**) The levels of p-JNK, p-ERK and Fra-1 in SDS22-knockdown or SDS22-overexpressing cells were analyzed by western blot. (**d**) The levels of p-JNK, p-ERK and Fra-1 in miR-134-transfected cells. (**e**) The levels of p-JNK, p-ERK and Fra-1 in cells co-transfected with miR-con/miR-134 and pcDNA3.1/pSDS22 as indicated. (**f**) Relative AP-1 activity in SDS22-knockdown or SDS22-overexpressing cells. (**g**) Relative AP-1 activity in T29 cells co-transfected with miR-con/miR-134 and pcDNA3.1/pSDS22 as indicated. (**h**) The levels of miR-134 in SDS22-knockdown T29 cells and SDS22-overexpressing T29H cells. (**i**) Fra-1 expression was induced in SKOV3 cells transfected with miR-134 mimics. (**j**) The relative levels of pri-miR-134 in SKOV3 cells transfected with miR-134 or siFRA-1. (**k**) The relative luciferase activity of the P1 constructs in miR-134- or siFRA-1-transfected SKOV3 cells. Data are shown as mean±S.D. from three independent experiments. *P<0.05, **P<0.01 by Student's *t*-test

Using three bioinformatics algorithms (TargetScan, PicTar and miRDB), we identified SDS22, TCF21 and PPP1R12A as putative miR-134 targets (Supplementary Figures S3a and b). To validate these findings, we cloned the 3′-untranslated region (3′-UTR) of SDS22, TCF21 and PPP1R12A into a luciferase reporter plasmid (Supplementary Figure S3c). In cells co-transfected with miR-134 mimics and the reporter constructs, only the reporter containing the wild type SDS22 3′-UTR was significantly inhibited by miR-134 (Figure 2a; Supplementary Figure S3d). Furthermore, endogenous SDS22 protein levels were reduced in cells transfected with miR-134 mimics and increased in cells transfected with miR-134 inhibitors (Figure 2b). However, neither the miR-134 mimics nor the miR-134 inhibitors significantly affected SDS22 mRNA levels (Supplementary Figure S3e). Together, these data indicate that SDS22 is a direct target of miR-134.

### miR-134 drives a positive feedback loop that amplifies ERK/JNK-AP-1 signaling

Given that JNK and ERK are substrates of protein phosphatase-1 (PP1)^32, 33^ and mammalian SDS22 was initially identified as an inhibitory subunit of PP1,^34, 35^ we hypothesized that miR-134 enhances PP1 activity by repressing SDS22 expression, thereby decreasing p-JNK and p-ERK levels. Surprisingly, we found that SDS22 knockdown increased p-JNK and p-ERK levels, whereas SDS22 overexpression reduced p-JNK and p-ERK levels (Figure 2c). In addition, miR-134 overexpression increased p-JNK and p-ERK levels (Figure 2d), and this effect was abolished by ectopic SDS22 expression (Figure 2e). These data indicate that SDS22 functions as a positive regulator of PP1 with respect to the dephosphorylation of p-JNK and p-ERK. JNK and ERK phosphorylation enhances AP-1 activity.^18, 19^ Consistently, SDS22 knockdown significantly enhanced Fra-1 levels and AP-1 activity, and SDS22 overexpression reduced Fra-1 levels and AP-1 activity (Figures 2c and f). In addition, miR-134 overexpression increased Fra-1 levels and AP-1 activity, and this effect was abolished by ectopic SDS22 expression (Figures 2d, e and g). Moreover, SDS22 knockdown increased miR-134 expression, and SDS22 overexpression inhibited miR-134 expression (Figure 2h). Furthermore, miR-134 mimics transfection increased Fra-1 and pri-miR-134 levels, and enhanced the activity of miR-134 promoter, and these effects were partially blocked by Fra-1 knockdown (Figures 2i–k). On the basis of these observations, we concluded that miR-134 mediates a positive feedback loop that amplifies ERK/JNK-AP-1 signaling by targeting SDS22.

### miR-134 promotes H2AX S139 phosphorylation

**Figure 3:**
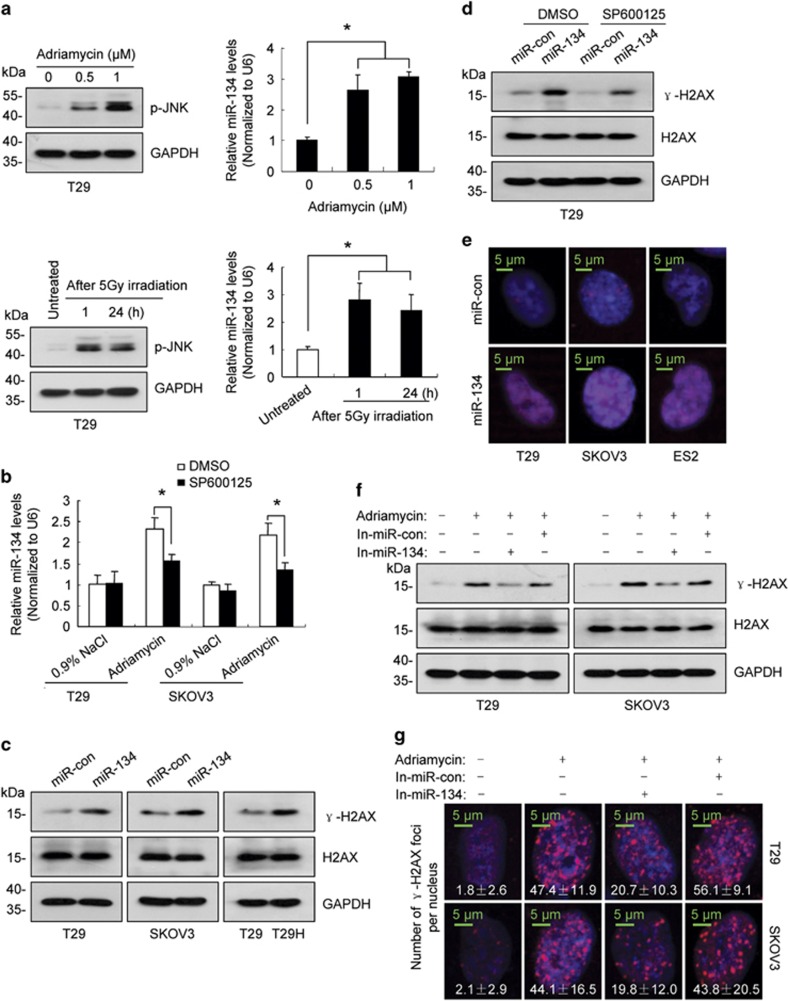
miR-134 regulates H2AX S139 phosphorylation by activating JNK. (**a**) The levels of p-JNK (left) and miR-134 (right) in T29 cells treated with adriamycin for 24 h or with radiation. (**b**) SP600125 partially abolished miR-134 upregulation induced by a 24 h of adriamycin (0.5 *μ*M) treatment. (**c**) Western blot revealed that *γ*-H2AX levels were elevated in cells transfected with miR-134 mimics. (**d**) Representative western blot images of *γ*-H2AX and H2AX in miR-134-expressing T29 cells or control cells treated with SP600125 or DMSO. (**e**) miR-134 induced an increase in *γ*-H2AX throughout the nucleus. (**f**) Representative western blot images of *γ*-H2AX and H2AX in miR-134 inhibitors-transfected cells after a 24 h of adriamycin (0.5 *μ*M) treatment. (**g**) Representative fluorescent images of *γ*-H2AX foci in miR-134 inhibitors-transfected cells after a 5 h of adriamycin (0.5 *μ*M) treatment, and the number of *γ*-H2AX foci per nucleus are indicated. Data are shown as mean±S.D. from three independent experiments. *P<0.05 by Student's *t*-test

Given that DNA damage activates JNK,^36^ we investigated whether DNA damage induces miR-134 expression. As shown in Figure 3a and Supplementary Figure S4, treatment with DNA damaging agents, including ionizing radiation, adriamycin and etoposide, increased p-JNK levels and upregulated miR-134 expression. Moreover, we demonstrated that treatment with the JNK inhibitor SP600125 blocked adriamycin-induced upregulation of miR-134 (Figure 3b). DNA damage leads to phosphorylation of H2AX at serine 139, and this modified form of H2AX is referred to as *γ*-H2AX.^37^ In addition to ataxia telangiectasia mutated (ATM), ATM- and Rad3-related and DNA-dependent protein kinase, JNK can also phosphorylate H2AX.^38^ Given that miR-134 overexpression increased JNK phosphorylation (Figures 2d and e), we investigated whether miR-134 regulates *γ*-H2AX. As shown in Figure 3c, ectopic miR-134 expression increased the levels of *γ*-H2AX, which was partially inhibited by SP600125 treatment (Figure 3d). In contrast to DNA damaging agents, which induce *γ*-H2AX foci formation, miR-134 increased *γ*-H2AX accumulation throughout the nucleus (Figure 3e). Finally, we investigated the role of miR-134 in H2AX S139 phosphorylation following DNA damage. As shown in Figures 3f and g, the miR-134 inhibitors partially blocked the DNA damage-induced increase of *γ*-H2AX.

### miR-134 enhances DNA repair by promoting H2AX S139 phosphorylation

**Figure 4:**
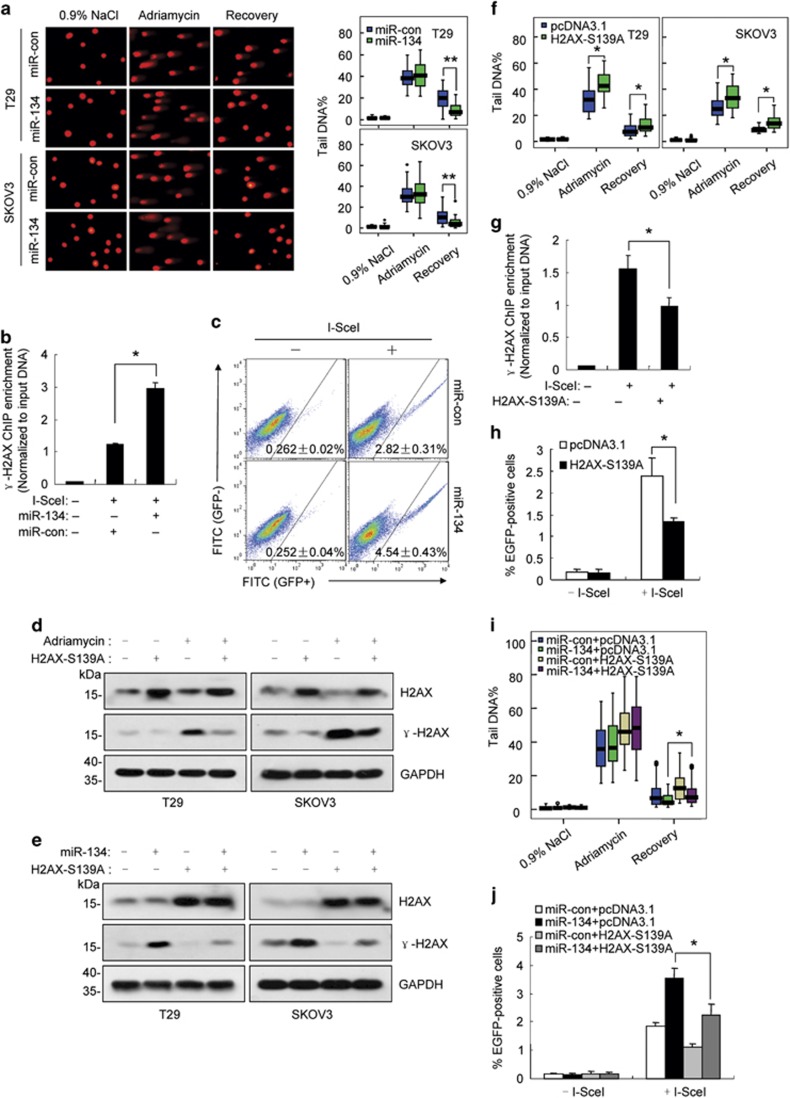
miR-134 enhances DNA repair efficiency by promoting H2AX S139 phosphorylation. (**a**) Left, representative comet assay revealing the formation of DNA breaks (formation of a ‘comet tail') in miR-134-transfected T29 and SKOV3 cells treated with adriamycin (0.5 *μ*M) for 24 h and then allowed to recover for 12 h. Right, box plot graph showing Tail DNA%. (**b**) ChIP assay with anti-*γ*-H2AX was performed 24 h after SKOV3-Hygro-EGFP cells were co-transfected with miR-134 or the control mimics. The genomic locations of the qPCR primers are referred to as ChIP primers in Supplementary Figure S6a. (**c**) The proportion of EGFP-positive cells was determined 48 h after SKOV3-Hygro-EGFP cells were co-transfected with the pCBASceI plasmid and miR-134 or the control mimics. (**d**) Representative western blot images of *γ*-H2AX and H2AX in H2AX-S139A-expressing cells or control cells 24 h after adriamycin (0.5 *μ*M) treatment. (**e**) Representative western blot images of *γ*-H2AX and H2AX in cells co-transfected with the H2AX-S139A plasmid and miR-134 or the control mimics for 48 h. (**f**) The box plot graph showing tail DNA% in H2AX-S139A-transfected cells treated with adriamycin (0.5 *μ*M) for 24 h and then allowed to recover for 12 h. (**g**) ChIP assay using anti-*γ*-H2AX was performed 24 h after SKOV3-Hygro-EGFP cells were co-transfected with pCBASceI and the H2AX-S139A plasmid or the control plasmid. (**h**) The proportion of EGFP-positive cells was determined 48 h after SKOV3-Hygro-EGFP cells were co-transfected with the pCBASceI plasmid and the H2AX-S139A plasmid or the control plasmid. (**i**) The box plot graph depicting tail DNA% in T29 cells co-transfected with miR-con/miR-134 and pcDNA3.1/H2AX-S139A after a 24 h adriamycin treatment and a 12-h recovery. (**j**) The proportion of EGFP-positive cells was determined 48 h after SKOV3-Hygro-EGFP cells were co-transfected with miR-con/miR-134 and pcDNA3.1/H2AX-S139A. Data are shown as mean±S.D. from three independent experiments. *P<0.05, **P<0.01 by Student's *t*-test

DNA damage repair is considered to be the primary function of *γ*-H2AX.^39, 40^ Therefore, we evaluated whether miR-134 promotes DNA repair. The neutral comet assay, which specifically detects DNA double-strand breaks (DSBs), suggested that miR-134 may facilitate DNA repair in adriamycin or etoposide-treated cells (Figure 4a; Supplementary Figure S5). As non-homologous end joining (NHEJ) is the predominant DSB repair pathway in mammalian cells,^41^ we investigated the effect of miR-134 on NHEJ-mediated repair. To this end, we developed an NHEJ assay similar to that reported previously (Supplementary Figure S6a).^42^ The pSceI-Hygro-EGFP plasmid, which contains 2 I-SceI endonuclease recognition sites in the reverse orientation, was integrated into the chromosomal DNA of SKOV3 cells and used as a substrate for DSB formation and subsequent NHEJ repair. Given that no I-SceI site is present in the human genome, transient transfection of the I-SceI expression plasmid specifically cleaves the substrate at I-SceI sites to generate DSBs. NHEJ of the two broken DNA strands results in the deletion of the hygromycin B phosphotransferase (HPH) open reading frame and enables enhanced green fluorescent protein (EGFP) translation. The efficiency of DSB formation was defined as the proportion of uncut DNA, and the efficiency of NHEJ was defined as the proportion of joined DNA and the percentage of EGFP-positive cells (Supplementary Figures S6b–d). As shown in Figures 4b and c, miR-134 overexpression increased *γ*-H2AX enrichment at DSB sites and enhanced NHEJ efficiency.

To confirm that increased the enhancement of DNA repair induced by miR-134 was a result of elevated *γ*-H2AX, we substituted serine 139 of H2AX with alanine (H2AX-S139A). H2AX-S139A expression partially blocked the increase in *γ*-H2AX levels in adriamycin-treated or miR-134 mimic-transfected cells (Figures 4d and e). The comet assay revealed that H2AX-S139A expression sensitized cells to adriamycin-induced DNA damage (Figure 4f). Moreover, ectopic H2AX-S139A expression led to a reduction of *γ*-H2AX at DSB sites and decreased NHEJ efficiency (Figures 4g and h). Most importantly, we demonstrated that the miR-134-mediated increase in DNA repair and NHEJ efficiency was partially blocked by ectopic H2AX-S139A expression (Figures 4i and j).

### miR-134 reduces chemosensitivity *in vitro* and *in vivo*

**Figure 5:**
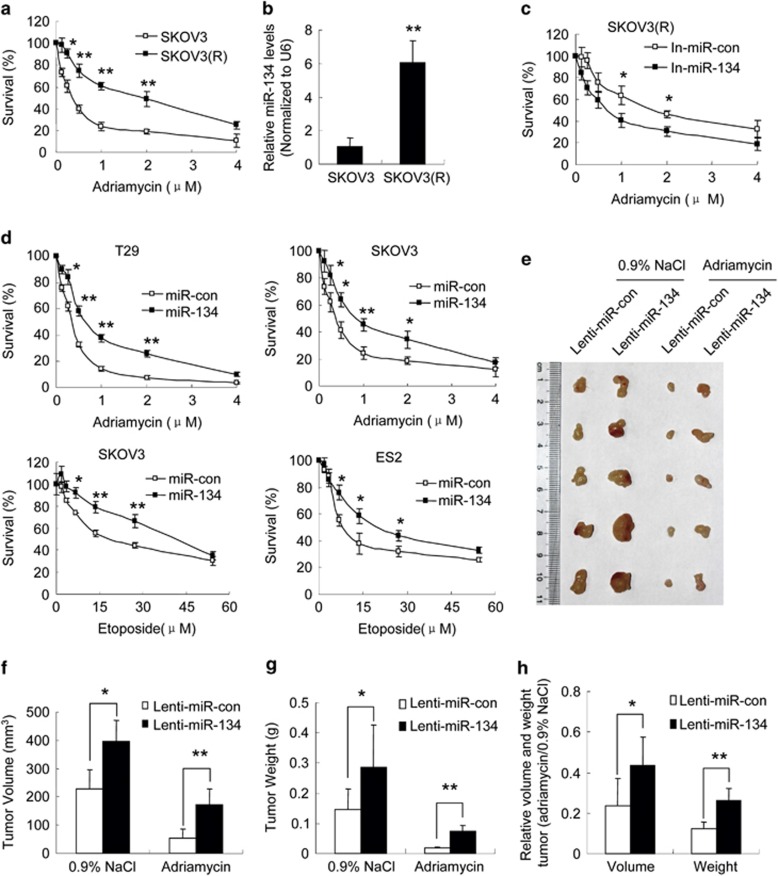
miR-134 reduces chemosensitivity *in vitro* and *in vivo.* (**a**) Cell proliferation assay was performed 48 h after adriamycin treatment. Survival (%) is expressed as the percentage of control cells. (**b**) miR-134 levels in SKOV3 and SKOV3(R) cells. (**c**) Cell proliferation assay in miR-134 inhibitors-transfected SKOV3(R) cells after 48 h of adriamycin treatment. (**d**) Cell proliferation assay of miR-con or miR-134-transfected cells treated with adriamycin or etoposide for 48 h. (**e–h**) Subcutaneous xenografts of SKOV3 cells infected with the miR-134 lentivirus or the control lentivirus were treated with adriamycin or 0.9% NaCl (*n*=5). (**e**) Images of the xenograft tumors. (**f**) The average volume of tumors. (**g**) The average weight of tumors. (**h**) The relative volume and weight of tumors treated with adriamycin or 0.9% NaCl. Data are shown as mean±S.D. from three independent experiments, unless noted otherwise. *P<0.05, **P<0.01 by Student's *t*-test

The observation that miR-134 enhances DNA repair led us to hypothesize that miR-134 overexpression might reduce sensitivity to DNA damaging agents. To test this hypothesis, we generated an adriamycin-resistant human ovarian cell line SKOV3(R) by selecting SKOV3 cells treated with increasing concentrations of adriamycin in a step-wise manner (Figure 5a). We found that miR-134 was upregulated in SKOV3(R) cells compared with SKOV3 cells (Figure 5b), and that treatment with the miR-134 inhibitors increased the sensitivity of SKOV3(R) cells to adriamycin (Figure 5c). Furthermore, treatment with adriamycin or etoposide strongly inhibited cell proliferation, and the miR-134 mimics diminished this effect (Figure 5d). To evaluate the effects of miR-134 on the sensitivity of ovarian tumors to DNA damaging agents *in vivo*, we established xenografts from SKVO3 cells infected with the miR-134 lentivirus or the control lentivirus in athymic nude (NU/NU) mice. As expected, miR-134 overexpression promoted tumor growth (Figures 5e–g). Moreover, treatment with adriamycin significantly inhibited tumor growth (Figures 5e–g). Most interestingly, adriamycin inhibited tumor growth to a lesser extent in the xenografts overexpressing miR-134 compared with the control (Figures 5e–h), indicating that miR-134 decreases chemosensitivity in ovarian cancer cells.

### miR-134 promotes cell proliferation, migration and invasion and enhances tumor growth

**Figure 6:**
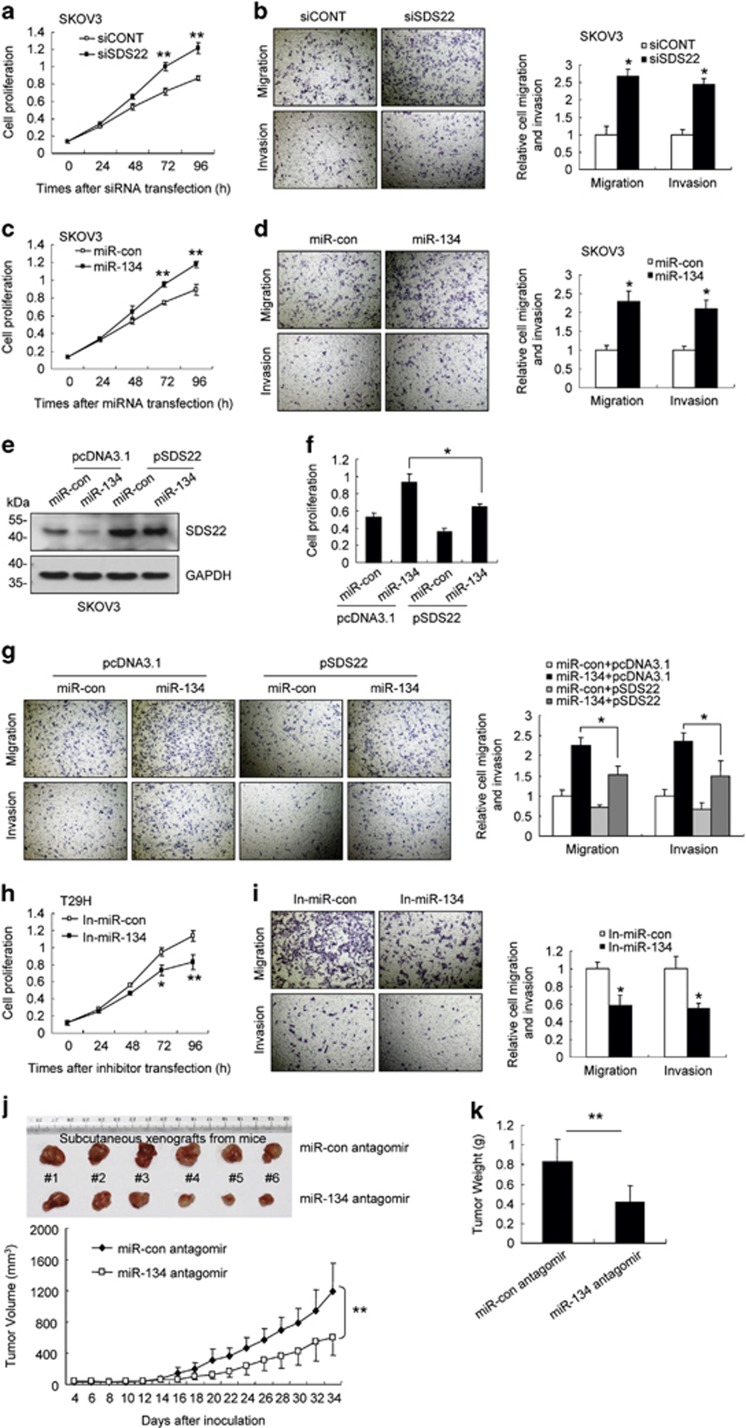
miR-134 promotes cell proliferation, migration and invasion, and enhances tumor growth. (**a** and **b**) SKOV3 cells were transfected with siSDS22 or siCONT. (**a**) The cell proliferation assay was performed at the indicated time points. (**b**) Representative micrographs of cell migration and invasion assays (left) and the quantification (right). (**c** and **d**) SKOV3 were transfected with miR-134 or the control mimics. (**c**) The cell proliferation assay was performed at the indicated time points. (**d**) Representative micrographs of cell migration and invasion assays (left) and the quantification (right). (**e–g**) SKOV3 cells were co-transfected with miR-con/miR-134 and pcDNA3.1/pSDS22 as indicated. (**e**) SDS22 expression was detected using western blot assays. (**f**) Cell proliferation was measured 96 h after transfection. (**g**) Representative micrographs of cell migration and invasion assays (left) and the quantification (right). (**h** and **i**) T29H cells were transfected with In-miR-134 or In-miR-con. (**h**) The cell proliferation assay was performed at the indicated time points. (**i**) Representative micrographs of cell migration and invasion assays (left) and the quantification (right). (**j** and **k**) Subcutaneous xenografts of T29H cells transfected with the miR-134 antagomir or the control antagomir (*n*=6). (**j**) The images of the tumors at autopsy are presented in the top panel, and the tumor volumes were measured at the indicated time period in the graph below. (**k**) The average weight of xenografted tumors was measured. Data are shown as mean±S.D. from three independent experiments, unless noted otherwise. *P<0.05, **P<0.01 by Student's *t*-test

SDS22 is a putative tumor suppressor gene in *Drosophila*, as loss of SDS22 facilitates Ras-mediated transformation by promoting tumor growth and metastasis.^43^ We found that SDS22 knockdown promoted proliferation, migration and invasion of T29 and SKOV3 cells (Figures 6a and b; Supplementary Figures S7a and b). In addition, miR-134 overexpression promoted cell proliferation, migration and invasion (Figures 6c and d; Supplementary Figures S7c and d), and this effect was partially blocked by ectopic SDS22 expression (Figures 6e–g and Supplementary Figures S7e–g). In contrast, transfection with the miR-134 inhibitors suppressed cell proliferation, migration and invasion in T29H cells (Figures 6h and i). Moreover, the xenografts derived from T29H cells transfected with the miR-134 antagomir grew at a significantly slower rate compared with the negative control xenografts (Figures 6j and k). Consistently, the levels of SDS22 were increased, and the levels of p-JNK, p-ERK and Fra-1 were reduced in the xenografts from miR-134 antagomir-transfected cells compared with the negative control xenografts (Supplementary Figure S8). Together, these data suggest that miR-134 promotes cell proliferation, migration and invasion by targeting SDS22.

### miR-134 is upregulated in ovarian cancer tissues and associated with poor outcomes in ovarian cancer patients

**Figure 7:**
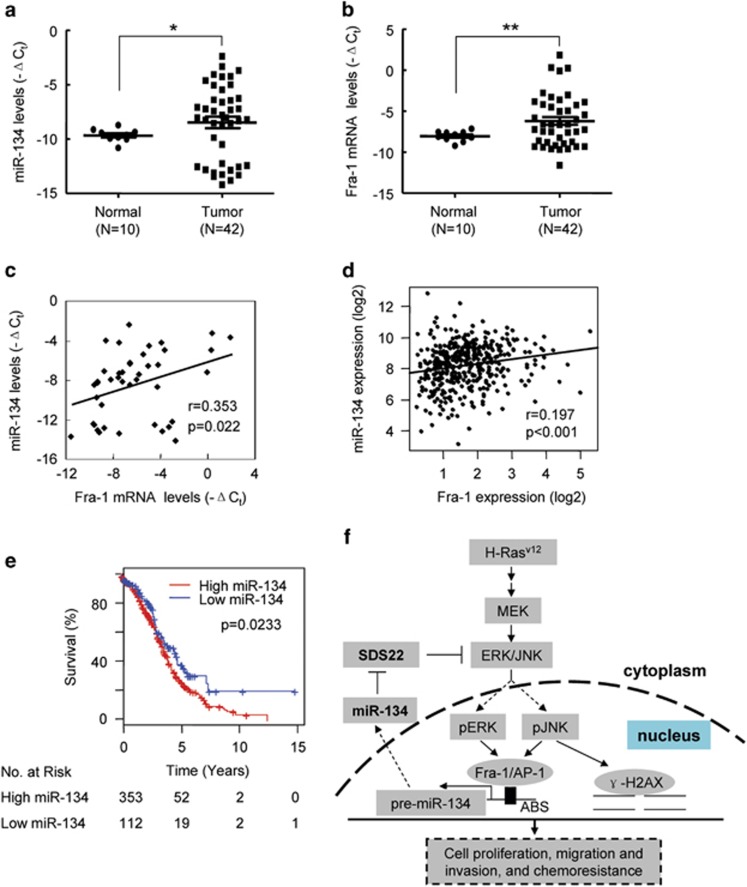
miR-134 is upregulated in ovarian cancer tissues and is associated with the outcomes of ovarian cancer patients. (**a** and **b**) The relative levels of miR-134 (**a**) and Fra-1 mRNA (**b**) in human primary ovarian cancer specimens derived from 42 ovarian cancer patients and 10 normal ovarian tissues. (**c**) The correlation of miR-134 and Fra-1 relative expression levels in 42 ovarian cancer samples. (**d**) The correlation of miR-134 and Fra-1 expression in 408 primary ovarian cancer samples. The data regarding the expression levels of miR-134 and Fra-1 were downloaded from the TCGA database. (**e**) Kaplan–Meier survival curves for ovarian cancer patients according to the miR-134 expression levels in tumor tissues, and significance was calculated using the log-rank test. (**f**) Schematic diagram of the Fra-1/miR-134/SDS22 feedback loop and its function in tumor development and chemoresistance. ABS, AP-1 binding sites. (**a** and **b**) Mean±S.E.M. are provided. *P<0.05, **P<0.01 by Student's *t*-test. (**c** and **d**) Spearman correlation coefficient with the respective significance is indicated

We examined miR-134 and Fra-1 expression in 42 human primary ovarian cancer specimens and 10 normal ovarian tissues.^44^ Real-time RT-PCR analysis demonstrated that miR-134 and Fra-1 mRNA expression was increased in human ovarian tumor tissues compared with normal ovarian tissues (Figures 7a and b), and Fra-1 expression levels were positively correlated with miR-134 expression levels (Figure 7c). The data downloaded from The Cancer Genome Atlas (TCGA) database revealed that the correlation between miR-134 and Fra-1 was also observed in 408 human ovarian tumor tissues (Figure 7d). As chemoresistance might reduce the overall survival of cancer patients, we investigated whether miR-134 expression levels were associated with overall survival by analyzing data from 465 ovarian patients downloaded from the TCGA database. As expected, the median overall survival was substantially reduced in patients expressing high levels of miR-134 compared with patients expressing low levels of miR-134 (3.65 *versus* 4.60 years, respectively, *P*=0.0233; Figure 7e).

## Discussion

In this study, we demonstrated that oncogenic H-Ras activated JNK and ERK, leading to the Fra-1-dependent upregulation of miR-134. miR-134 promoted the activation of JNK and ERK by targeting SDS22. As a result, JNK and ERK induced Fra-1 expression, which increased the activity of the miR-134 promoter and upregulated miR-134 expression. Moreover, we demonstrated that miR-134 augmented H2AX S139 phosphorylation by activating JNK and promoted DNA repair, thereby reducing chemosensitivity. Furthermore, we revealed that miR-134 promotes cell proliferation, migration and invasion and enhances tumor growth. Interestingly, miR-134 was upregulated in ovarian cancer tissues, and miR-134 expression in tumor tissues was inversely correlated with the median overall survival of ovarian cancer patients. Together, these data suggest that a miR-134-mediated positive feedback loop amplifies ERK/JNK signaling and promotes chemoresistance (Figure 7f).

Consistent with previous reports that miR-134 has oncogenic activity in head and neck carcinoma and in lung adenocarcinoma,^9, 10, 11^ we demonstrated that miR-134 acts as an oncogene to amplify the ERK/JNK signaling pathways in ovarian cancer cells. However, these findings contradict a previous report that miR-134 functions as a tumor suppressor by targeting K-Ras in renal carcinoma cells.^15^ Analysis of data from the TCGA database indicated that higher expression levels of miR-134 were associated with decreased median overall survival in 10 types of cancers, but were associated with increased median overall survival in three types of cancers (Supplementary Table S2a). Therefore, the oncogenic or tumor suppressive activity exhibited by miR-134 may depend on the cell context and the genetic background of the tumor.

Chemotherapy is a standard approach for the treatment of various tumors. However, many patients experience tumor recurrence due to treatment failure resulting from chemoresistance. miR-134 sensitizes cancer cells to chemotherapy by various mechanisms.^12, 45, 46^ miR-134 sensitizes MCF-7/ADR cells to doxorubicin by downregulating ABCC1.^45^ It also enhances the sensitivity of SKOV3-TR30 cells to paclitaxel by targeting Pak2 and promoting apoptosis.^46^ However, miR-134 overexpression confers resistance to the EGFR inhibitor gefitinib.^11^ In the current study, we found that miR-134 was upregulated in the adriamycin-resistant human ovarian cell line SKOV3(R) compared with the parental SKOV3 cell line. In addition, we demonstrated that miR-134 decreases chemosensitivity by enhancing DNA repair. Aberrant activation of ERK signaling mediates resistance to EGFR kinase inhibitors,^47, 48^ and we demonstrated here that miR-134 promoted ERK phosphorylation. Therefore, our findings might shed light on the molecular basis underlying the observation that miR-134 overexpression confers resistance to the EGFR inhibitor gefitinib.^11^

We demonstrated that oncogenic Ras induced miR-134 upregulation in ovarian cancer cells. In addition, the expression of miR-134 in 42 human primary ovarian cancer specimens was upregulated compared with normal ovarian tissues. However, an oncogenic Ras mutation was only observed in 1 out of the 42 human primary ovarian cancer specimens evaluated (data not shown). A survey of the cBioPortal and TCGA database revealed that only 4 out of the 307 ovarian cancer tissues harbored oncogenic Ras mutations (Supplementary Table S2b). Our findings suggest that the upregulation of miR-134 in ovarian cancer tissues is not a consequence of oncogenic Ras mutations. Interestingly, we found that Fra-1 is required for miR-134 upregulation in ovarian cancer cells. In addition, the expression of miR-134 was positively correlated with Fra-1 expression in ovarian cancer tissues. TCGA database analysis revealed that the expression of Fra-1 was positively correlated with miR-134 expression in 20 out of 30 types of cancers, including ovarian cancer (Supplementary Table S2c). Taken together, these data suggest that Fra-1 has a pivotal role in regulating miR-134 expression in these tumors.

The primary function of *γ*-H2AX is believed to be associated with DNA damage repair.^39, 40^ Although *γ*-H2AX is dispensable for the initial recruitment of repair factors to DSB sites, it is required for the accumulation of repair factors to radiation-induced foci.^49^ H2AX(−/−) cells or H2AX(−/−) cells that express mutant H2AX-S139A were hypersensitive to DSB-inducing reagents, such as *γ*-rays, etoposide and temozolamide.^39^ In this study, ectopic H2AX-S139A expression inhibited the phosphorylation of endogenous H2AX following DNA damaging treatment and repressed NHEJ-mediated DNA repair. Moreover, miR-134 promoted H2AX S139 phosphorylation by amplifying JNK signaling and promoting DNA repair, and these activities were partially blocked by ectopic H2AX-S139A expression. Interestingly, miR-134 induced a pan-nuclear increase of *γ*-H2AX, indicating that the increase in *γ*-H2AX levels is not due to increased proliferation, which leads to *γ*-H2AX foci formation by increasing the number of active replicons.^40, 50^ We propose that the pan-nuclear increase of *γ*-H2AX induced by miR-134 might facilitate *γ*-H2AX foci formation upon DNA damage, subsequently recruiting DNA damage response factors to DNA damage sites and promoting DNA repair.

Although SDS22 was identified as an inhibitory subunit of PP1 in rat liver nuclei,^34^ we demonstrated that SDS22 functions as a positive regulator of PP1 with respect to p-JNK and p-ERK dephosphorylation. This is not surprising given that SDS22 promotes the dephosphorylation activity of PP1 on Aurora B.^51^ In addition, *S. pombe* SDS22 was initially identified as a positive regulator of PP1.^52^ Together, these findings suggest that the regulation of PP1 by SDS22 is dependent on the specific substrate.^35^ SDS22 is a tumor suppressor gene in *Drosophila*, and loss of SDS22 promotes tumor growth and metastasis in Ras^V12^ cells.^43^ SDS22 is frequently deleted in breast, cervical, esophageal squamous, lung, melanoma, ovarian and renal cancers.^43, 53, 54^ SDS22 is also significantly downregulated in multiple cancers.^43, 55^ Consistent with these reports, we found that SDS22 suppressed ovarian cancer cell proliferation, invasion and migration. However, the analysis of 42 human primary ovarian cancer specimens and 10 normal ovarian tissues revealed that SDS22 mRNA expression was only slightly downregulated in ovarian cancer tissues (data not shown), and a survey of the TCGA database indicated that SDS22 mRNA levels were not associated with the overall survival of ovarian cancer patients (Supplementary Table S2a). Interestingly, the SDS22 protein levels were reduced in ovarian cancer tissues, in which miR-134 was upregulated (Supplementary Figure S9). So we demonstrated that miR-134 inhibited SDS22 at the protein level rather than the mRNA levels. Therefore, further efforts are needed to determine whether SDS22 protein levels are associated with overall survival in ovarian cancer patients.

In summary, we found a Fra-1-miR-134-SDS22 positive feedback loop that amplifies ERK/JNK signaling and reduces chemosensitivity in ovarian cancer cells. Our findings suggest that manipulating the expression of miR-134 or its target genes may improve chemotherapy efficacy in ovarian cancer.

## Materials and Methods

### Cell culture, clinical specimens and drug treatment

The human ovarian epithelial cell lines T29 and T29H were kindly provided by Dr. Jinsong Liu^27, 28^ and maintained in DMEM medium (Hyclone, Logan, UT, USA). Human HEK293T, SKOV3 and ES2 cell lines were acquired from ATCC (Manassas, VA, USA). An adriamycin-resistant human ovarian cell line SKOV3(R) by selecting SKOV3 cells treated with increasing concentrations of adriamycin in a step-wise manner. HEK293T and SKOV3 cells were grown in RPMI 1640 medium (Hyclone), ES2 cells were grown in McCoy's 5A medium (Life Technologies, Grand Island, NY, USA), all supplemented with 10% fetal bovine serum and incubated at 37 °C in a humidified incubator with 5% CO_2_. Cell line authentication is achieved by genetic profiling using polymorphic short tandem repeat loci. The 42 primary human ovarian tumors and 10 normal tissues used in the present study were previously described.^44^ Informed consent was obtained from all patients for the collection and use of clinical samples, and the study was approved by the Scientific Ethics Committee of The General Hospital of the People's Liberation Army and Wenzhou Medical University. Cells were treated with U0126, SP600125 or FTI-277 (Sigma, Saint Louis, MO, USA) at a final concentration of 10 *μ*M for 24 h before subsequent treatment or analysis. For DNA damaging treatments, cells were exposed to adriamycin or etoposide for 24 h before subsequent analysis, or as specifically indicated.

### Plasmids

To construct the miR-134 promoter reporter plasmids (P1, P2 and P3), three genomic fragments in the 4 kb region upstream of the pre-miR-134 gene were inserted into the NheI/BglII sites of the pGL6-TA vector (Beyotime, Shanghai, China). The 3′-UTRs reporter plasmids (SDS22 3′-UTR, TCF21 3′-UTR, PPP1R12A 3′-UTR) were generated by cloning the 3′-UTR sequences into the pGL3-control vector (Promega, Madison, WI, USA) at the Xba1 site. To generate the mutant SDS22 reporter (SDS22-mut 3′-UTR), the seed region of the SDS22 3′-UTR was mutated using the QuikChange II XL mutagenesis kit (Agilent Technologies, La Jolla, CA, USA). The coding sequence of SDS22 was amplified and cloned into pcDNA3.1 (Life Technologies) to generate SDS22 expressing plasmid lacking the 3′-UTR (pSDS22). To construct the pSceI-Hygro-EGFP plasmid, the HPH open reading frame, which is flanked by 2 I-SceI endonuclease recognition sites in the reverse orientation, was cloned into the pEGFP-N1 vector (Clontech, Mountain View, CA, USA) at *Hin*dIII/*Bam*HI sites. The coding sequence of H2AX was amplified and cloned into pcDNA3.1 to construct the H2AX expressing plasmid H2AX(WT). The mutant H2AX expressing plasmid (H2AX-S139A) was generated using the QuikChange II XL mutagenesis kit (Agilent Technologies). The pAP1-TA-luc and pRL-CMV plasmids were purchased from Beyotime and Promega, respectively. The pCBASceI plasmid was obtained from Addgene (Cambridge, MA, USA). The primers used for cloning are provided in Supplementary Table S3.

### miRNA microarray

Total RNA was extracted from T29 and T29H cells using TRIzol reagent (Life Technologies). miRNA microarray was performed as previously described.^56^ All data were deposited into the Gene Expression Omnibus (GSE70416).

### miRNA, siRNA and transfection

miRNAs (miR-134 mimics, miR-134 inhibitors and the corresponding negative controls) were obtained from RiboBio (Guangzhou, China) and referred to as miR-134, miR-con, In-miR-134 and In-miR-con, respectively. siRNAs were obtained from Life Technologies (Shanghai, China). miRNA and siRNA were transfected at a final concentration of 50 nM using Lipofectamine 2000 Reagent (Life Technologies) according to the manufacturer's instruction. The cells were then collected 48 h after transfection for subsequent analysis.

### Real-time RT-PCR

Total RNA was prepared using TRIzol reagent (Life Technologies) and incubated with RNase-free DNase I (Promega) for 30 min. To quantify mRNA levels, the DNA-free RNA was reverse transcribed using the M-MLV reverse transcription kit (Promega) according to the manufacturer's instructions. Samples prepared in the absence of reverse transcriptase served as negative controls. Real-time RT-PCR was performed using SYBR premix Ex Taq (TaKaRa, Dalian, China) and the ABI 7300 Sequence Detection System (Life Technologies). GAPDH mRNA expression levels served as an internal control. The sequences of the qPCR primers are listed in Supplementary Table S3. To evaluate miRNA expression, reverse transcription and real-time RT-PCR were performed using the bulge-loop miRNA qPCR primer set (RiboBio) according to the manufacturer's instructions, and the data were normalized to the expression level of human U6 small nuclear RNA.

### Western blot

Western blot analysis was performed as described previously.^57^ The primary antibody anti-Fra-1 (sc-28310) was obtained from Santa Cruz Biotechnology (Santa Cruz, CA, USA). Anti-SDS22 (TA500614) was obtained from OriGene Technologies (Beijing, China). Anti-H-Ras (05-775) was obtained from Millipore. Anti-GAPDH (#2118), anti-p-ERK (#9101), anti-ERK (#9102), anti-p-JNK (#9251), anti-*γ*-H2AX (#2577) and anti-H2AX (#2595) were obtained from Cell Signaling Technology (Danvers, MA, USA).

### Luciferase assays

Cells were co-transfected with 0.4 *μ*g of the firefly luciferase reporter vector and 0.02 *μ*g of the *Renilla* luciferase control vector (pRL-CMV) using Lipofectamine 2000 in a 24-well plate. Luciferase assays were performed 48 h after transfection using the dual-luciferase reporter assay system (Promega). Firefly luciferase activity was normalized to the *Renilla* luciferase activity.

### Chromatin immunoprecipitation

ChIP assays were performed as described previously.^26^ The anti-Fra-1 (sc-28310) or the mouse IgG control (Active Motif, Carlsbad, CA, USA), anti-*γ*-H2AX (AF2288, R&D Systems, Minneapolis, MN, USA) or the rabbit IgG control (R&D Systems) were used to precipitate DNA. The primers of ChIP-PCR are provided in Supplementary Table S3.

### *γ*-H2AX immunofluorescence

Immunofluorescence assays were performed as described previously^57^ and the anti-*γ*-H2AX antibody (AF2288, R&D Systems) was used.

### Neutral comet assays

Neutral comet assays were performed as described previously.^58^ Comet images were visualized using a fluorescence microscope (LEICA DMI3000 B).

### NHEJ assay in SKOV3 cells

The pSceI-Hygro-EGFP plasmid was transfected into SKOV3 cells using the Lipofectamine 2000 Reagent (Life Technologies). The hygromycin B (50 μg/ml)-resistant clones were transfected with the pCBASceI plasmid, and the EGFP-positive clones were referred to as SKOV3-Hygro-EGFP cells. NHEJ assay was performed by transfecting the pCBASceI plasmid into the SKOV3-Hygro-EGFP clones. NHEJ was assessed using fluorescence-activated cell sorting and qPCR analyses. The efficiency of NHEJ was calculated as the relative proportion of joined and uncut DNA, and the number of EGFP-positive cells at the indicated time points. Cells were trypsinized, washed with PBS and analyzed with a FACSCalibur fluorescence-activated cell sorter (Becton Dickinson, Franklin Lakes, NJ, USA). To quantify the levels of uncut and joined DNA, 20 ng of genomic DNA was analyzed using qPCR. The RPS20 promoter was used as an internal control. The primers used for qPCR are provided in Supplementary Table S3. To determine the effect of miR-134 or H2AX-S139A on NHEJ, SKOV3-Hygro-EGFP cells were pretreated with miR-134, H2AX-S139A or the negative control for 48 h and subsequently co-transfected with pCBASceI for 48 h and processed for flow cytometry analysis of EGFP.

### Animal studies

To evaluate the effect of miR-134 overexpression, SKOV3 cells were infected with a lentivirus expressing miR-134 or the negative control (Genechem, Shanghai, China). To evaluate the effect of miR-134 inhibition, T29H were transfected with the miR-134 antagomir or the negative control (RiboBio). For the xenograft studies, 6-week-old female athymic nude mice (nu/nu) were purchased from Vital River Experimental Animal Center (Beijing, China) and maintained under pathogen-free conditions. The miR-con- and miR-134-overexpressing SKOV3 cells or the control- and miR-134-antagomir transfected T29H cells were harvested and resuspended in PBS. A total of 2.5 × 10^6^ (SKOV3) or 5 × 10^6^ (T29H) cells in 100 *μ*l PBS were subcutaneously (s.c.) injected into the rear flank of the mice. Tumors were measured using a caliper, and tumor volume was calculated using the following: *v*=*L* × *W*^2^ × 0.5236 (*L*=long axis, *W*=short axis). For adriamycin treatment, when the tumor masses became visible, an intraperitoneal (i.p.) injection of adriamycin dissolved in 0.09% NaCl at a dose of 5 mg/kg was administered to the mice twice a week for 3 weeks. After 34 days, the mice were killed, and the tumors were harvested and weighed. All animals were maintained and treated in accordance with the guidelines of the Institutional Animal Care and Use Committee of Wenzhou Medical University.

### Cell proliferation assays

Cells (2 × 10^3^) were plated in 96-well plates and transfected with 5 pmol of miRNA (miR-134 and miR-con), In-miRNA (In-miR-134 and In-miR-con) or siRNA (siSDS22 and siCONT) using siPORT NeoFX (Life Technologies). Cell proliferation was evaluated using the CellTiter 96 AQueous One Solution Cell Proliferation Assay kit (Promega) according to the manufacturer's instruction.

### *In vitro* migration and invasion assays

Migration and invasion assays were conducted as we described previously,^26^ and 2.5 × 10^4^ cells and 2 × 10^5^ cells were used for 4 h migration and 24 h invasion, respectively.

### TCGA data sets analysis

TCGA expression data determined using HiSeq 2000 platform and clinical data were obtained from the TCGA Data Portal (http://cancergenome.nih.gov/). Ras mutation data were obtained from the cBioportal database (http://www.cbioportal.org/). miRNA and mRNA expression had been determined by next generation sequencing data using HiSeq 2000 platform. RPM was used to quantify miRNA expression levels from the miRNA-Seq datasets. mRNA expression was calculated as RPKM values in the ovarian cancer study and RSEM values in the studies of other tumors. The normalized values of miRNA and mRNA expression were converted to log_2_-transformed values. The relation between gene expression levels and survival was explored by separating the cases into two groups by the data-driven approach.^59^

### Statistical analysis

Data are presented as mean±S.D. or±S.E.M.. Unless noted otherwise, each experiment was carried out in triplicates. Differences were analyzed by a two-tailed Student's *t*-test. The correlation between two genes was analyzed by Pearson correlation algorithm. The univariate hazard ratio with 95% confidence interval was calculated using the Cox proportional hazards model, and significance was calculated using Wald's test. *P<*0.05 was considered statistically significant.

## Abbreviations

AP-1: activator protein-1
ATM: ataxia telangiectasia mutated
ChIP: chromatin immunoprecipitation
DSBs: DNA double-strand breaks
EGFP: enhanced green fluorescent protein
HPH: hygromycin B phosphotransferase
MAPK: mitogen-activated protein kinase
MEK: mitogen-activated protein kinase kinase
miRNAs: microRNAs
mRNAs: messenger RNAs
NHEJ: non-homologous end joining
p-ERK: phosphorylated extracellular signal-regulated kinase
p-JNK: phosphorylated c-Jun NH2 kinase
PP1: protein phosphatase-1
RT-PCR: real-time reverse transcription-polymerase chain reaction
3′-UTR: 3′-untranslated region
