# Supplementary material for: The Fra-1–miR-134–SDS22 feedback loop amplifies ERK/JNK signaling and reduces chemosensitivity in ovarian cancer cells
Source: Cell Death Dis. 2016 Sep 29;7(9):e2384–. doi: 10.1038/cddis.2016.289 (PMC5059884; doi:10.1038/cddis.2016.289)
Supplement: Supplementary Tables [file cddis2016289x1.doc]

**Supplementary Tables**

**Supplementary Table S1.** The expression of miRNAs in T29H and T29 cells.

**Supplementary Table S2a** The association between patient survival and the expression levels of miR-134, Fra-1 and SDS22 in patients with various cancers.

**Supplementary Table S2b** The frequency of Ras mutations in different tumor types.

**Supplementary Table S2c** Correlation of Fra-1 and miR-134 expression levels in different tumor tissues.

**Supplementary Table S3.** Oligonucleotides used for real-time RT-PCR, qChIP, plasmid construction and siRNA.

| **Supplementary Table S1 Expression of miRNAs in T29H and T29 cells** | | | | | |
| --- | --- | --- | --- | --- | --- |
| **ProbeSet Name** | **T29H-1** | **T29H-2** | **T29-1** | **T29-2** | **Fold change** |
| hsa-miR-127-3p_st | 135.3993 | 117.744 | 6.994535 | 18.82724 | 9.80 |
| hsa-miR-409-3p_st | 186.6122 | 166.2342 | 22.69302 | 18.75163 | 8.51 |
| hsa-miR-382_st | 66.58492 | 48.21157 | 15.03055 | 10.50284 | 4.50 |
| hsa-miR-379_st | 51.17294 | 58.51949 | 11.62561 | 17.65192 | 3.75 |
| hsa-miR-432_st | 66.68943 | 53.78264 | 17.53843 | 18.86803 | 3.31 |
| hsa-miR-31_st | 1480.827 | 1149.902 | 273.9697 | 536.5251 | 3.25 |
| hsa-miR-129-star_st | 200.3962 | 134.5247 | 100.4102 | 15.34506 | 2.89 |
| hsa-miR-431_st | 52.36538 | 29.10058 | 10.10521 | 18.26658 | 2.87 |
| hsa-miR-143_st | 43.85183 | 29.45471 | 13.976 | 11.93204 | 2.83 |
| hsa-miR-134_st | 64.59252 | 40.46675 | 18.83647 | 18.79934 | 2.79 |
| hsa-miR-520h_st | 31.40549 | 13.30935 | 9.538543 | 7.89007 | 2.57 |
| hsa-miR-34a_st | 33.84495 | 32.99419 | 11.16531 | 15.94209 | 2.47 |
| hsa-miR-935_st | 60.8692 | 64.04642 | 22.75034 | 29.48941 | 2.39 |
| hsa-miR-145_st | 42.86354 | 53.11963 | 15.85629 | 24.54747 | 2.38 |
| hsa-miR-29b-1-star_st | 32.01898 | 33.89422 | 16.963 | 11.9248 | 2.28 |
| hsa-miR-146a_st | 37.79827 | 34.48883 | 16.74639 | 15.76821 | 2.22 |
| hsa-miR-370_st | 16.96415 | 33.5252 | 11.96909 | 11.60153 | 2.14 |
| hsa-miR-21-star_st | 40.21196 | 30.2872 | 15.13099 | 19.04322 | 2.06 |
| hsa-miR-181a_st | 249.9611 | 199.7554 | 66.86448 | 153.0256 | 2.05 |
| hsa-let-7e_st | 652.2837 | 600.8638 | 201.9147 | 412.9014 | 2.04 |
| hsa-miR-125b_st | 687.305 | 691.106 | 235.7571 | 479.6144 | 1.93 |
| hsa-miR-27a-star_st | 28.80463 | 22.75079 | 12.38043 | 14.40907 | 1.92 |
| hsa-miR-154_st | 36.33709 | 20.51535 | 14.52024 | 15.33518 | 1.90 |
| hsa-miR-130b_st | 108.085 | 116.601 | 69.96283 | 49.63697 | 1.88 |
| hsa-miR-548c-3p_st | 14.77853 | 26.82053 | 14.59682 | 7.714893 | 1.86 |
| hsa-miR-606_st | 54.13544 | 50.32926 | 42.59934 | 13.73316 | 1.85 |
| hsa-miR-654-3p_st | 27.76104 | 31.58655 | 18.80166 | 14.11961 | 1.80 |
| hsa-miR-125b-1-star_st | 36.49496 | 38.37895 | 16.74569 | 25.08092 | 1.79 |
| hsa-miR-100_st | 540.5343 | 537.062 | 246.8107 | 362.8859 | 1.77 |
| hsa-miR-142-5p_st | 46.65094 | 20.57938 | 24.86931 | 13.28889 | 1.76 |
| hsa-miR-299-3p_st | 31.46104 | 25.92998 | 18.02993 | 14.88226 | 1.74 |
| hsa-miR-1308_st | 259.2734 | 175.7973 | 77.63188 | 175.6515 | 1.72 |
| hsa-miR-181b_st | 262.9759 | 257.671 | 114.3437 | 189.2699 | 1.71 |
| hsa-miR-125a-5p_st | 452.5355 | 373.4616 | 171.9651 | 313.4498 | 1.70 |
| hsa-miR-24-2-star_st | 31.01455 | 27.90153 | 25.23757 | 9.978575 | 1.67 |
| hsa-miR-499-3p_st | 20.88661 | 21.93044 | 14.36719 | 11.72775 | 1.64 |
| hsa-miR-493_st | 25.2789 | 21.10294 | 12.89988 | 15.93847 | 1.61 |
| hsa-miR-93-star_st | 53.81986 | 28.43871 | 28.08873 | 24.79095 | 1.56 |
| hsa-miR-654-5p_st | 21.87836 | 25.33346 | 12.11747 | 18.28341 | 1.55 |
| hsa-miR-495_st | 22.96963 | 28.49943 | 18.30742 | 15.59272 | 1.52 |
| hsa-miR-345_st | 94.1116 | 60.28622 | 36.94185 | 64.91668 | 1.52 |
| hsa-miR-574-3p_st | 191.669 | 176.8419 | 138.4191 | 106.2806 | 1.51 |
| hsa-miR-363_st | 39.18198 | 29.89755 | 31.90129 | 14.06331 | 1.50 |
| hsa-miR-1247_st | 20.41235 | 35.53021 | 19.13921 | 18.2332 | 1.50 |
| hsa-miR-182_st | 191.0362 | 135.6944 | 82.6061 | 136.1094 | 1.49 |
| hsa-miR-361-5p_st | 154.0845 | 152.867 | 90.3931 | 115.39 | 1.49 |
| hsa-miR-1825_st | 28.36157 | 25.6531 | 20.00921 | 16.42789 | 1.48 |
| hsa-miR-24_st | 1560.818 | 1439.75 | 688.6643 | 1350.908 | 1.47 |
| hsa-miR-584_st | 24.33949 | 19.27451 | 15.8149 | 13.89524 | 1.47 |
| hsa-miR-708_st | 26.68889 | 18.70965 | 15.03928 | 16.27194 | 1.45 |
| hsa-miR-151-5p_st | 325.4944 | 274.6122 | 106.7744 | 307.1656 | 1.45 |
| hsa-miR-1301_st | 23.09524 | 33.81398 | 23.47167 | 15.80086 | 1.45 |
| hsa-miR-665_st | 24.59445 | 31.37541 | 23.76418 | 14.94207 | 1.45 |
| hsa-miR-122-star_st | 29.01717 | 16.21901 | 15.96469 | 16.23908 | 1.40 |
| hsa-miR-642_st | 33.8712 | 19.61646 | 16.01955 | 22.5234 | 1.39 |
| hsa-miR-132_st | 31.96829 | 30.47108 | 23.98354 | 21.30907 | 1.38 |
| hsa-miR-378_st | 100.0042 | 70.04654 | 38.17083 | 85.35806 | 1.38 |
| hsa-miR-487a_st | 23.16079 | 36.48423 | 17.57634 | 25.85394 | 1.37 |
| hsa-miR-23b_st | 666.6219 | 664.3867 | 394.3865 | 581.5482 | 1.36 |
| hsa-miR-99b_st | 393.119 | 353.2634 | 274.2671 | 274.5633 | 1.36 |
| hsa-miR-767-5p_st | 18.56151 | 26.71437 | 8.843554 | 24.66024 | 1.35 |
| hsa-miR-641_st | 25.43641 | 15.5066 | 13.46008 | 16.96256 | 1.35 |
| hsa-miR-425-star_st | 20.43781 | 28.86011 | 18.18108 | 18.45632 | 1.35 |
| hsa-miR-320d_st | 166.6223 | 160.9754 | 66.22794 | 178.4007 | 1.34 |
| hsa-miR-625_st | 36.83239 | 36.50344 | 27.27753 | 29.16735 | 1.30 |
| hsa-miR-17-star_st | 19.30986 | 26.9344 | 14.53924 | 21.36712 | 1.29 |
| hsa-miR-376a_st | 17.77549 | 29.14548 | 16.80161 | 19.66997 | 1.29 |
| hsa-miR-155_st | 222.2547 | 177.8768 | 91.62466 | 219.9221 | 1.28 |
| hsa-miR-568_st | 26.43866 | 9.883478 | 13.71721 | 14.57063 | 1.28 |
| hsa-miR-1224-3p_st | 28.22044 | 31.42546 | 28.27079 | 18.59419 | 1.27 |
| hsa-miR-125a-3p_st | 37.61634 | 43.77903 | 29.81072 | 34.17976 | 1.27 |
| hsa-let-7a_st | 537.8216 | 501.6098 | 198.4459 | 619.3231 | 1.27 |
| hsa-miR-629_st | 36.38419 | 53.09417 | 32.70369 | 37.89933 | 1.27 |
| hsa-miR-596_st | 12.49432 | 26.57069 | 18.26337 | 12.77849 | 1.26 |
| hsa-let-7c_st | 265.7364 | 236.2881 | 100.5703 | 300.3743 | 1.25 |
| hsa-miR-196a-star_st | 29.70971 | 23.84063 | 26.0108 | 16.78709 | 1.25 |
| hsa-miR-154-star_st | 27.47343 | 19.55583 | 12.58337 | 25.22134 | 1.24 |
| hsa-miR-501-3p_st | 28.84205 | 29.37543 | 16.96511 | 29.85217 | 1.24 |
| hsa-miR-99a_st | 30.1445 | 64.73259 | 47.0556 | 29.40948 | 1.24 |
| hsa-miR-500_st | 24.38457 | 28.9775 | 17.82199 | 25.50623 | 1.23 |
| hsa-miR-1227_st | 43.32123 | 29.73856 | 31.21393 | 28.25424 | 1.23 |
| hsa-miR-210_st | 62.28663 | 62.1354 | 32.96504 | 68.60841 | 1.22 |
| hsa-miR-194_st | 29.442 | 22.03318 | 21.48677 | 20.82802 | 1.22 |
| hsa-miR-939_st | 27.59402 | 23.22383 | 19.58899 | 22.19979 | 1.22 |
| hsa-miR-193a-5p_st | 47.73081 | 41.27518 | 47.13757 | 26.23141 | 1.21 |
| hsa-miR-21_st | 65.59411 | 88.04681 | 36.79094 | 90.31898 | 1.21 |
| hsa-miR-337-3p_st | 29.33678 | 15.70859 | 15.2608 | 22.02033 | 1.21 |
| hsa-miR-1280_st | 97.50738 | 92.76019 | 53.86095 | 104.0116 | 1.21 |
| hsa-miR-652_st | 34.91768 | 36.57607 | 22.96157 | 37.0085 | 1.19 |
| hsa-miR-27a_st | 225.6917 | 173.3989 | 67.23183 | 268.6477 | 1.19 |
| hsa-miR-197_st | 96.14701 | 76.12415 | 72.5966 | 72.44477 | 1.19 |
| hsa-miR-22_st | 353.3293 | 296.7826 | 120.1145 | 428.1705 | 1.19 |
| hsa-miR-320c_st | 467.8294 | 423.2711 | 353.4659 | 405.0212 | 1.17 |
| hsa-miR-337-5p_st | 24.94541 | 37.83797 | 28.78728 | 25.49095 | 1.16 |
| hsa-miR-532-3p_st | 29.05627 | 45.40712 | 32.03902 | 32.37847 | 1.16 |
| hsa-miR-18a-star_st | 24.76214 | 30.75639 | 25.73077 | 22.31568 | 1.16 |
| hsa-miR-27b_st | 80.4187 | 75.09886 | 34.19632 | 100.4497 | 1.16 |
| hsa-miR-365_st | 25.57916 | 17.0985 | 21.2467 | 15.78986 | 1.15 |
| hsa-miR-625-star_st | 29.9987 | 31.32414 | 31.58578 | 21.71643 | 1.15 |
| hsa-miR-320b_st | 580.4248 | 481.6774 | 466.262 | 457.3329 | 1.15 |
| hsa-miR-1181_st | 17.88596 | 21.03025 | 18.77019 | 15.20527 | 1.15 |
| hsa-miR-23a_st | 1154.945 | 1261.209 | 621.7675 | 1491.068 | 1.14 |
| hsa-miR-185_st | 79.65844 | 102.9215 | 51.86953 | 107.9346 | 1.14 |
| hsa-miR-532-5p_st | 33.86395 | 22.21632 | 25.29461 | 24.16916 | 1.13 |
| hsa-miR-151-3p_st | 80.71207 | 68.76299 | 38.78461 | 93.06882 | 1.13 |
| hsa-miR-886-3p_st | 131.4322 | 81.84672 | 64.11729 | 124.3001 | 1.13 |
| hsa-miR-335-star_st | 25.93007 | 14.54232 | 16.23372 | 19.74564 | 1.12 |
| hsa-miR-1255a_st | 22.57407 | 26.93439 | 23.8537 | 20.37295 | 1.12 |
| hsa-miR-1184_st | 20.72237 | 53.97584 | 38.91391 | 27.99996 | 1.12 |
| hsa-miR-1225-3p_st | 20.20623 | 30.91927 | 21.21345 | 24.63451 | 1.12 |
| hsa-miR-335_st | 332.8286 | 461.3983 | 665.3861 | 47.59838 | 1.11 |
| hsa-miR-129-3p_st | 28.43143 | 33.45256 | 34.99426 | 20.82802 | 1.11 |
| hsa-miR-500-star_st | 43.09636 | 37.12714 | 31.58576 | 41.02206 | 1.10 |
| hsa-miR-923_st | 1798.38 | 1577.94 | 1286.723 | 1805.985 | 1.09 |
| hsa-miR-324-3p_st | 29.62129 | 33.58077 | 29.57848 | 28.32696 | 1.09 |
| hsa-miR-615-5p_st | 20.20533 | 29.41988 | 20.13495 | 25.37319 | 1.09 |
| hsa-miR-638_st | 212.0068 | 199.8868 | 150.9613 | 229.2172 | 1.08 |
| hsa-miR-140-3p_st | 99.27795 | 118.0056 | 64.31887 | 136.4678 | 1.08 |
| hsa-miR-720_st | 95.58956 | 83.32771 | 60.8802 | 105.9803 | 1.07 |
| hsa-let-7b_st | 933.2152 | 903.4259 | 748.7042 | 967.0605 | 1.07 |
| hsa-miR-152_st | 45.89981 | 40.91327 | 26.58018 | 54.5801 | 1.07 |
| hsa-miR-296-5p_st | 36.16949 | 38.68654 | 28.61575 | 41.54921 | 1.07 |
| hsa-miR-1826_st | 5137.796 | 4780.131 | 4198.468 | 5122.403 | 1.06 |
| hsa-miR-483-3p_st | 19.97552 | 28.48746 | 23.98354 | 21.84138 | 1.06 |
| hsa-miR-331-3p_st | 23.96492 | 17.66838 | 13.52976 | 25.92069 | 1.06 |
| hsa-miR-1281_st | 24.58922 | 27.17438 | 29.20386 | 20.13268 | 1.05 |
| hsa-miR-362-5p_st | 24.34221 | 23.10074 | 24.23512 | 21.03698 | 1.05 |
| hsa-let-7i_st | 375.9778 | 321.5001 | 238.8606 | 427.1161 | 1.05 |
| hsa-miR-222_st | 1627.405 | 1640.448 | 1092.124 | 2029.114 | 1.05 |
| hsa-miR-744_st | 86.60008 | 84.49849 | 64.7308 | 98.98356 | 1.05 |
| hsa-miR-425_st | 49.74516 | 39.78319 | 25.25269 | 60.43287 | 1.04 |
| hsa-miR-23a-star_st | 16.3629 | 22.73263 | 19.39852 | 18.08016 | 1.04 |
| hsa-miR-320a_st | 514.8053 | 466.7841 | 510.322 | 432.398 | 1.04 |
| hsa-miR-1234_st | 29.68011 | 55.55355 | 36.13911 | 45.74938 | 1.04 |
| hsa-miR-551b-star_st | 14.23236 | 24.82302 | 22.70941 | 14.90606 | 1.04 |
| hsa-miR-888_st | 15.836 | 32.91444 | 31.43746 | 15.63423 | 1.04 |
| hsa-miR-92b-star_st | 28.60743 | 36.46403 | 37.00956 | 25.85715 | 1.04 |
| hsa-miR-1244_st | 21.80181 | 25.31893 | 23.40019 | 22.34467 | 1.03 |
| hsa-miR-29a_st | 155.8228 | 141.4651 | 73.65415 | 216.3871 | 1.02 |
| hsa-miR-324-5p_st | 61.49319 | 65.30553 | 39.15753 | 85.0145 | 1.02 |
| hsa-miR-221_st | 1146.606 | 1104.009 | 755.9191 | 1453.31 | 1.02 |
| hsa-let-7g_st | 36.64102 | 42.8014 | 32.13924 | 46.91116 | 1.00 |
| hsa-miR-765_st | 19.82027 | 21.80396 | 28.73903 | 13.09454 | 0.99 |
| hsa-miR-7-2-star_st | 22.18189 | 16.3893 | 22.42741 | 16.38945 | 0.99 |
| hsa-miR-421_st | 26.67313 | 23.70439 | 19.65552 | 31.07263 | 0.99 |
| hsa-miR-574-5p_st | 42.55803 | 52.5358 | 38.92755 | 57.09595 | 0.99 |
| hsa-miR-944_st | 15.90794 | 17.07901 | 13.13496 | 20.30878 | 0.99 |
| hsa-miR-548i_st | 22.70426 | 12.35641 | 16.25657 | 19.52473 | 0.98 |
| hsa-miR-106b_st | 151.6434 | 153.7268 | 76.14494 | 236.3658 | 0.98 |
| hsa-miR-1285_st | 27.44979 | 21.42223 | 22.30297 | 27.73838 | 0.98 |
| hsa-miR-877-star_st | 19.70669 | 24.70193 | 27.70807 | 18.13199 | 0.97 |
| hsa-miR-1183_st | 18.08857 | 25.09962 | 25.92798 | 18.85408 | 0.96 |
| hsa-miR-1274a_st | 25.67106 | 23.77084 | 24.53193 | 26.84898 | 0.96 |
| hsa-miR-296-3p_st | 29.52371 | 35.87004 | 29.18242 | 39.07405 | 0.96 |
| hsa-miR-126_st | 20.59797 | 22.23507 | 11.25215 | 33.46586 | 0.96 |
| hsa-let-7d_st | 528.1129 | 396.4793 | 355.2276 | 611.0942 | 0.96 |
| hsa-miR-552_st | 26.59408 | 11.57489 | 15.84613 | 24.14066 | 0.95 |
| hsa-miR-494_st | 657.5741 | 486.4869 | 394.4783 | 810.2615 | 0.95 |
| hsa-miR-214_st | 28.29734 | 16.99268 | 26.53007 | 21.34442 | 0.95 |
| hsa-miR-553_st | 15.64506 | 19.43345 | 21.96348 | 15.21521 | 0.94 |
| hsa-miR-149-star_st | 188.7467 | 173.3775 | 189.1804 | 195.868 | 0.94 |
| hsa-miR-183_st | 15.63387 | 25.34955 | 19.12589 | 24.58825 | 0.94 |
| hsa-miR-663_st | 40.17922 | 46.08339 | 36.91626 | 55.39992 | 0.93 |
| hsa-miR-208b_st | 21.95452 | 33.3397 | 39.54969 | 19.92015 | 0.93 |
| hsa-miR-604_st | 13.52164 | 23.4396 | 28.62709 | 11.1872 | 0.93 |
| hsa-miR-503_st | 43.05921 | 62.15902 | 44.79932 | 68.96338 | 0.92 |
| hsa-miR-940_st | 16.06112 | 27.80545 | 26.18864 | 21.41347 | 0.92 |
| hsa-miR-107_st | 421.9372 | 376.239 | 306.6308 | 566.8053 | 0.91 |
| hsa-miR-559_st | 29.49498 | 20.09202 | 24.10577 | 30.16486 | 0.91 |
| hsa-miR-502-3p_st | 29.62424 | 24.1783 | 29.48282 | 29.40947 | 0.91 |
| hsa-miR-16-2-star_st | 15.97014 | 20.50247 | 24.74007 | 15.32487 | 0.91 |
| hsa-miR-505-star_st | 26.09021 | 21.23677 | 29.76354 | 22.49776 | 0.91 |
| hsa-miR-193b_st | 138.361 | 117.1772 | 98.76817 | 184.0893 | 0.90 |
| hsa-miR-106b-star_st | 23.75246 | 38.17572 | 26.01981 | 42.65956 | 0.90 |
| hsa-miR-877_st | 40.31159 | 32.25277 | 49.64247 | 30.89948 | 0.90 |
| hsa-miR-1249_st | 38.86757 | 33.53236 | 28.86579 | 51.55123 | 0.90 |
| hsa-miR-575_st | 15.31718 | 19.5232 | 25.99968 | 12.81209 | 0.90 |
| hsa-miR-1180_st | 28.56855 | 25.67075 | 31.45775 | 28.96679 | 0.90 |
| hsa-miR-563_st | 17.06215 | 28.95045 | 37.23362 | 14.25908 | 0.89 |
| hsa-miR-1246_st | 42.19634 | 91.97874 | 109.1475 | 41.27108 | 0.89 |
| hsa-miR-30a-star_st | 25.1146 | 41.19261 | 32.70368 | 41.77357 | 0.89 |
| hsa-miR-1207-5p_st | 150.164 | 175.3372 | 162.483 | 203.9652 | 0.89 |
| hsa-miR-18a_st | 129.5788 | 101.1004 | 77.67854 | 187.1526 | 0.87 |
| hsa-miR-191_st | 314.4873 | 285.6712 | 339.5309 | 349.9953 | 0.87 |
| hsa-miR-637_st | 19.40455 | 32.55008 | 40.21922 | 19.89919 | 0.86 |
| hsa-miR-187-star_st | 22.76491 | 18.79651 | 27.89509 | 20.34292 | 0.86 |
| hsa-miR-938_st | 22.45083 | 24.36913 | 23.80641 | 30.57053 | 0.86 |
| hsa-miR-1237_st | 27.19357 | 22.71727 | 30.50149 | 27.52005 | 0.86 |
| hsa-miR-181a-2-star_st | 47.1001 | 52.37363 | 74.07501 | 42.06377 | 0.86 |
| hsa-miR-518b_st | 25.9995 | 15.83521 | 17.69567 | 31.45207 | 0.85 |
| hsa-miR-423-3p_st | 146.3919 | 146.9732 | 127.2602 | 218.5039 | 0.85 |
| hsa-miR-373-star_st | 13.60437 | 34.28664 | 36.66083 | 19.8814 | 0.85 |
| hsa-miR-1274b_st | 25.7766 | 26.78167 | 22.74185 | 39.36845 | 0.85 |
| hsa-miR-99b-star_st | 31.33707 | 38.96145 | 45.41482 | 37.87276 | 0.84 |
| hsa-miR-378-star_st | 19.2526 | 14.92525 | 16.54822 | 24.05054 | 0.84 |
| hsa-miR-92a_st | 538.9954 | 459.3395 | 453.2635 | 735.8766 | 0.84 |
| hsa-miR-93_st | 455.0724 | 354.6235 | 389.5934 | 575.4116 | 0.84 |
| hsa-miR-25_st | 80.09225 | 86.36563 | 46.97417 | 152.0288 | 0.84 |
| hsa-miR-103_st | 513.1954 | 397.374 | 462.3713 | 632.2017 | 0.83 |
| hsa-miR-629-star_st | 40.1097 | 21.5399 | 51.00167 | 23.15036 | 0.83 |
| hsa-miR-455-3p_st | 81.72311 | 105.4065 | 92.39087 | 133.1052 | 0.83 |
| hsa-miR-1228-star_st | 57.54148 | 93.6051 | 86.61951 | 96.34239 | 0.83 |
| hsa-miR-10b_st | 30.08669 | 30.6949 | 37.39458 | 36.28275 | 0.82 |
| hsa-miR-30e_st | 22.17905 | 23.96247 | 45.75203 | 10.22479 | 0.82 |
| hsa-miR-645_st | 26.84483 | 18.31592 | 37.85289 | 17.55938 | 0.81 |
| hsa-miR-579_st | 17.50318 | 14.16151 | 11.26746 | 27.72841 | 0.81 |
| hsa-miR-631_st | 17.23774 | 17.88693 | 32.27516 | 11.00742 | 0.81 |
| hsa-miR-768-5p_st | 66.44519 | 53.61919 | 55.52375 | 92.49711 | 0.81 |
| hsa-miR-555_st | 18.33728 | 23.51635 | 30.21852 | 21.51211 | 0.81 |
| hsa-miR-20a_st | 199.0648 | 167.3032 | 113.6264 | 340.9197 | 0.81 |
| hsa-miR-15b_st | 172.9066 | 169.104 | 112.2589 | 314.4463 | 0.80 |
| hsa-miR-576-3p_st | 137.0931 | 161.0033 | 357.9793 | 14.64478 | 0.80 |
| hsa-miR-106a_st | 334.273 | 273.5786 | 225.8968 | 538.1239 | 0.80 |
| hsa-miR-339-5p_st | 50.66648 | 32.00549 | 40.73019 | 63.23265 | 0.80 |
| hsa-miR-634_st | 18.49937 | 26.40085 | 28.72628 | 28.20145 | 0.79 |
| hsa-miR-1268_st | 67.9663 | 85.62054 | 91.40968 | 104.481 | 0.78 |
| hsa-miR-886-5p_st | 119.9393 | 99.75808 | 97.33443 | 183.0459 | 0.78 |
| hsa-miR-671-5p_st | 33.00874 | 37.65779 | 30.69917 | 59.60446 | 0.78 |
| hsa-miR-92b_st | 74.21296 | 60.86469 | 67.90141 | 105.074 | 0.78 |
| hsa-miR-28-5p_st | 48.44568 | 49.30645 | 35.70457 | 90.29928 | 0.78 |
| hsa-miR-302e_st | 16.27641 | 12.67359 | 7.623127 | 29.69855 | 0.78 |
| hsa-miR-17_st | 325.7941 | 306.9011 | 271.9967 | 545.8878 | 0.77 |
| hsa-miR-548c-5p_st | 5.174562 | 28.25886 | 28.49515 | 14.85379 | 0.77 |
| hsa-miR-1229_st | 29.24332 | 25.36443 | 37.21974 | 34.05702 | 0.77 |
| hsa-miR-1271_st | 15.22928 | 20.09201 | 31.99714 | 14.46178 | 0.76 |
| hsa-miR-192-star_st | 16.2446 | 14.65911 | 14.12548 | 26.55469 | 0.76 |
| hsa-miR-622_st | 20.98321 | 71.6892 | 87.37885 | 34.74145 | 0.76 |
| hsa-miR-29b-2-star_st | 16.48146 | 25.90411 | 18.68884 | 37.55219 | 0.75 |
| hsa-miR-193b-star_st | 14.1977 | 21.8112 | 33.73325 | 14.1196 | 0.75 |
| hsa-miR-1238_st | 24.76107 | 12.89458 | 31.47536 | 18.68685 | 0.75 |
| hsa-miR-149_st | 47.50898 | 68.65247 | 65.58747 | 90.66949 | 0.74 |
| hsa-miR-133b_st | 13.61297 | 16.43209 | 14.50772 | 25.96606 | 0.74 |
| hsa-miR-936_st | 12.78104 | 18.23379 | 21.9416 | 20.20916 | 0.74 |
| hsa-miR-25-star_st | 21.19161 | 27.86769 | 32.4501 | 34.23707 | 0.74 |
| hsa-miR-1253_st | 16.66535 | 15.12696 | 14.5967 | 28.67301 | 0.73 |
| hsa-miR-339-3p_st | 13.47532 | 22.47439 | 22.12741 | 26.9246 | 0.73 |
| hsa-miR-19b_st | 109.5105 | 45.81364 | 42.12878 | 170.493 | 0.73 |
| hsa-miR-589-star_st | 27.25912 | 15.23178 | 19.19513 | 39.23469 | 0.73 |
| hsa-miR-491-5p_st | 29.65582 | 22.13622 | 29.9113 | 41.54303 | 0.72 |
| hsa-miR-484_st | 20.00159 | 30.2872 | 24.1694 | 45.50882 | 0.72 |
| hsa-miR-1236_st | 21.91867 | 18.32332 | 23.37707 | 32.56811 | 0.72 |
| hsa-miR-744-star_st | 29.13983 | 27.3369 | 55.00613 | 23.53267 | 0.72 |
| hsa-miR-26a_st | 404.7697 | 397.8834 | 365.7326 | 756.0316 | 0.72 |
| hsa-miR-1202_st | 14.81905 | 13.8719 | 27.92584 | 12.27608 | 0.71 |
| hsa-miR-548j_st | 18.97996 | 30.41305 | 48.61203 | 20.89351 | 0.71 |
| hsa-miR-20b_st | 29.06869 | 25.53556 | 25.59982 | 51.42546 | 0.71 |
| hsa-miR-609_st | 18.15924 | 15.61656 | 37.13322 | 11.03879 | 0.70 |
| hsa-miR-1269_st | 15.43865 | 25.31367 | 39.86945 | 18.43581 | 0.70 |
| hsa-miR-1275_st | 44.50797 | 68.48833 | 75.38911 | 86.28352 | 0.70 |
| hsa-miR-224_st | 24.83425 | 18.23116 | 29.09003 | 32.74948 | 0.70 |
| hsa-miR-1307_st | 17.65111 | 29.96596 | 25.43841 | 43.09775 | 0.69 |
| hsa-miR-921_st | 10.91604 | 21.83067 | 20.13753 | 27.51706 | 0.69 |
| hsa-miR-362-3p_st | 17.90647 | 16.85139 | 38.41882 | 12.17781 | 0.69 |
| hsa-miR-147b_st | 13.49459 | 17.59108 | 34.87591 | 11.28127 | 0.67 |
| hsa-miR-342-3p_st | 145.7664 | 256.4441 | 531.4015 | 66.40899 | 0.67 |
| hsa-miR-423-5p_st | 41.22734 | 40.43074 | 48.85316 | 74.29837 | 0.66 |
| hsa-miR-1260_st | 23.2123 | 30.90972 | 34.50557 | 48.01191 | 0.66 |
| hsa-miR-192_st | 22.29788 | 21.83084 | 43.0909 | 24.37506 | 0.65 |
| hsa-miR-768-3p_st | 82.73346 | 46.20618 | 106.9498 | 95.70992 | 0.64 |
| hsa-miR-379-star_st | 7.892058 | 19.32844 | 27.80772 | 15.49737 | 0.63 |
| hsa-miR-361-3p_st | 12.67585 | 16.359 | 14.52064 | 32.09458 | 0.62 |
| hsa-miR-138-1-star_st | 24.76137 | 39.93037 | 55.90979 | 49.45904 | 0.61 |
| hsa-miR-487b_st | 212.4319 | 355.7352 | 822.1909 | 103.2754 | 0.61 |
| hsa-miR-28-3p_st | 32.08926 | 30.94974 | 26.9473 | 76.26192 | 0.61 |
| hsa-miR-591_st | 11.18466 | 11.27093 | 12.68433 | 24.11396 | 0.61 |
| hsa-miR-10a_st | 53.03917 | 71.10823 | 117.0087 | 86.66073 | 0.61 |
| hsa-miR-598_st | 12.55904 | 28.03421 | 57.20893 | 10.91695 | 0.60 |
| hsa-miR-424-star_st | 35.14043 | 45.45793 | 52.11766 | 84.06493 | 0.59 |
| hsa-miR-130a_st | 81.96033 | 71.97662 | 59.866 | 200.8725 | 0.59 |
| hsa-miR-194-star_st | 14.69455 | 17.03559 | 36.94234 | 17.05165 | 0.59 |
| hsa-miR-1287_st | 8.344151 | 15.47443 | 14.14822 | 26.40608 | 0.59 |
| hsa-miR-1254_st | 15.10474 | 19.62947 | 28.82865 | 30.45352 | 0.59 |
| hsa-miR-656_st | 10.41207 | 11.57488 | 27.26512 | 10.45374 | 0.58 |
| hsa-miR-889_st | 20.64045 | 18.28655 | 50.4966 | 16.34665 | 0.58 |
| hsa-miR-30c_st | 267.1901 | 795.6353 | 1394.949 | 433.7312 | 0.58 |
| hsa-miR-1231_st | 10.36332 | 19.61552 | 32.61991 | 19.25691 | 0.58 |
| hsa-miR-219-2-3p_st | 16.35972 | 28.40593 | 69.78297 | 8.726728 | 0.57 |
| hsa-miR-181d_st | 20.13151 | 19.10496 | 29.78629 | 39.06271 | 0.57 |
| hsa-miR-885-5p_st | 17.86123 | 11.87346 | 31.31778 | 21.17316 | 0.57 |
| hsa-miR-657_st | 18.8308 | 16.37426 | 39.68612 | 22.53988 | 0.57 |
| hsa-miR-548a-3p_st | 51.25127 | 103.0264 | 265.9276 | 10.20922 | 0.56 |
| hsa-miR-15a_st | 27.87571 | 30.61298 | 35.8856 | 70.13874 | 0.55 |
| hsa-let-7g-star_st | 11.58511 | 10.76905 | 13.27768 | 27.30136 | 0.55 |
| hsa-miR-215_st | 22.22569 | 17.08611 | 56.09952 | 15.41723 | 0.55 |
| hsa-miR-16_st | 450.0942 | 404.7713 | 396.3619 | 1172.3 | 0.54 |
| hsa-miR-564_st | 10.44374 | 17.15945 | 29.88548 | 20.92453 | 0.54 |
| hsa-miR-30a_st | 88.91502 | 193.7738 | 431.5371 | 105.5585 | 0.53 |
| hsa-miR-615-3p_st | 21.42122 | 36.71067 | 39.69701 | 72.50049 | 0.52 |
| hsa-miR-585_st | 14.56891 | 38.2883 | 85.48908 | 17.69964 | 0.51 |
| hsa-miR-483-5p_st | 16.84908 | 43.15417 | 84.60695 | 34.45929 | 0.50 |
| hsa-miR-1250_st | 11.94172 | 21.69083 | 33.68922 | 33.28088 | 0.50 |
| hsa-miR-181c-star_st | 284.5135 | 1352.29 | 2960.137 | 324.3565 | 0.50 |
| hsa-miR-30b_st | 206.9326 | 521.3959 | 1267.067 | 208.1889 | 0.49 |
| hsa-miR-653_st | 28.557 | 32.08449 | 91.70331 | 38.70322 | 0.47 |
| hsa-miR-181a-star_st | 218.1214 | 726.2728 | 1836.208 | 208.1669 | 0.46 |
| hsa-let-7f_st | 71.68593 | 99.66505 | 297.1135 | 77.31802 | 0.46 |
| hsa-miR-885-3p_st | 14.9698 | 41.27516 | 112.0504 | 15.17188 | 0.44 |
| hsa-miR-766_st | 15.28908 | 20.73994 | 59.84112 | 25.46832 | 0.42 |
| hsa-miR-132-star_st | 244.7557 | 716.8578 | 2149.102 | 163.2316 | 0.42 |
| hsa-miR-34b_st | 16.94762 | 25.79355 | 81.35556 | 21.43025 | 0.42 |
| hsa-miR-941_st | 12.82518 | 18.40684 | 26.40772 | 51.15288 | 0.40 |
| hsa-miR-220a_st | 25.21278 | 38.17039 | 145.0839 | 18.81688 | 0.39 |
| hsa-miR-374a-star_st | 97.11855 | 268.6566 | 944.7477 | 49.76058 | 0.37 |
| hsa-miR-454-star_st | 63.03525 | 294.5194 | 992.3034 | 64.56138 | 0.34 |
| hsa-miR-1827_st | 41.06836 | 33.88101 | 221.8021 | 12.26533 | 0.32 |
| hsa-miR-411_st | 32.71112 | 119.2606 | 494.2495 | 36.9451 | 0.29 |
| hsa-miR-30d_st | 40.10508 | 92.93421 | 398.6442 | 82.86543 | 0.28 |
| hsa-miR-1293_st | 32.06483 | 29.07848 | 228.6807 | 13.89951 | 0.25 |
| hsa-miR-1288_st | 19.40812 | 136.5005 | 914.4808 | 73.45116 | 0.16 |

| **Supplementary Table S2a Association analysis of patient survival with the expression levels of miR-134, Fra-1 amd SDS22 in different cancer patients** | | | | | | | | | | | |
| --- | --- | --- | --- | --- | --- | --- | --- | --- | --- | --- | --- |
| **Cancer Types** | **miR-134** | | |  | **Fra-1** | | |  | **SDS22** | | |
|  | **HR(95% CI)** | **P-value** | **Censoring** |  | **HR(95% CI)** | **P-value** | **Censoring** |  | **HR(95% CI)** | **P-value** | **Censoring** |
| Acute Myeloid Leukemia(LAML) | 0.62 (0.40-0.98) | **0.04** | 38.89% |  | 1.79 (1.15-2.80) | 0.01 | 38.93% |  | 2.09 (1.38-3.17) | 0.00 | 38.93% |
| Adrenocortical carcinoma(ACC) | 2.12 (0.94-4.75) | 0.07 | 67.50% |  | 3.75 (1.49-9.46) | 0.01 | 68.35% |  | 2.10 (0.84-5.27) | 0.11 | 68.35% |
| Bladder urothelial carcinoma(BLCA) | 2.25 (1.45-3.50) | 0.00 | 72.90% |  | 1.40 (0.85-2.29) | 0.18 | 72.73% |  | 0.46 (0.28-0.74) | 0.00 | 72.73% |
| Brain Lower Grade Glioma(LGG) | 2.17 (1.34-3.51) | 0.00 | 83.10% |  | 3.61 (1.79-7.27) | 0.00 | 82.98% |  | 0.44 (0.27-0.72) | 0.00 | 82.98% |
| Breast invasive carcinoma(BRCA) | 1.41 (0.85-2.34) | 0.18 | 89.59% |  | 0.57 (0.33-0.97) | 0.04 | 88.84% |  | 0.66 (0.43-1.01) | 0.06 | 88.84% |
| Cervical and endocervical cancers(CESC) | 1.29 (0.77-2.16) | 0.33 | 75.72% |  | 1.62 (0.92-2.84) | 0.10 | 75.52% |  | 0.69 (0.37-1.29) | 0.25 | 75.52% |
| Cholangiocarcinoma(CHOL) | 0.52 (0.16-1.66) | 0.27 | 56.25% |  | 2.71 (0.59-12.36) | 0.20 | 56.25% |  | 2.12 (0.69-6.54) | 0.19 | 56.25% |
| Colon adenocarcinoma(COAD) | 1.97 (0.82-4.76) | 0.13 | 81.77% |  | 1.46 (0.82-2.61) | 0.20 | 83.54% |  | 1.47 (0.84-2.55) | 0.17 | 83.54% |
| Esophageal carcinoma(ESCA) | 2.23 (1.28-3.91) | 0.00 | 55.46% |  |  |  |  |  |  |  |  |
| Glioblastoma multiforme(GBM) |  |  |  |  | 2.12 (1.31-3.43) | 0.00 | 35.62% |  | 1.56 (1.01-2.40) | 0.05 | 35.62% |
| Head and Neck squamous cell carcinoma(HNSC) | 1.76 (1.15-2.68) | 0.01 | 66.81% |  | 2.28 (1.44-3.62) | 0.00 | 66.60% |  | 1.36 (0.97-1.92) | 0.08 | 66.60% |
| Kidney Chromophobe(KICH) | 3.48 (0.87-13.94) | 0.08 | 87.30% |  | 0.24 (0.03-1.92) | 0.18 | 87.30% |  | 0.32 (0.08-1.37) | 0.12 | 87.30% |
| Kidney renal clear cell carcinoma(KIRC) | 1.23 (0.87-1.73) | 0.23 | 67.37% |  | 2.00 (1.46-2.74) | 0.00 | 68.02% |  | 1.38 (0.99-1.90) | 0.05 | 68.02% |
| Kidney renal papillary cell carcinoma(KIRP) | 4.86 (2.27-10.42) | 0.00 | 87.04% |  | 2.10 (0.89-4.95) | 0.09 | 86.98% |  | 0.41 (0.19-0.88) | 0.02 | 86.98% |
| Liver hepatocellular carcinoma(LIHC) | 1.95 (1.15-3.32) | 0.01 | 72.18% |  | 1.42 (0.91-2.20) | 0.12 | 71.63% |  | 1.60 (0.84-3.03) | 0.15 | 71.63% |
| Lung adenocarcinoma(LUAD) | 1.73 (1.01-2.96) | 0.05 | 72.25% |  | 2.22 (1.54-3.21) | 0.00 | 71.32% |  | 0.63 (0.42-0.96) | 0.03 | 71.32% |
| Lung squamous cell carcinoma(LUSC) | 1.37 (0.89-2.10) | 0.15 | 66.14% |  | 1.30 (0.94-1.81) | 0.12 | 63.38% |  | 1.25 (0.90-1.74) | 0.19 | 63.38% |
| Lymphoid Neoplasm Diffuse Large B-cell Lymphoma(DLBC) | 0.27 (0.03-2.64) | 0.26 | 86.49% |  | 3.05 (0.32-29.40) | 0.33 | 86.84% |  | 0.31 (0.04-2.24) | 0.25 | 86.84% |
| Mesothelioma(MESO) | 0.80 (0.44-1.44) | 0.46 | 22.67% |  | 3.13 (1.80-5.43) | 0.00 | 22.67% |  | 0.46 (0.25-0.84) | 0.01 | 22.67% |
| **Ovarian serous cystadenocarcinoma(OV)** | **1.42 (1.05-1.93)** | **0.02** | **44.73%** |  | 0.79 (0.56-1.10) | 0.16 | 44.17% |  | 0.83 (0.60-1.14) | 0.24 | 44.17% |
| Pancreatic adenocarcinoma(PAAD) | 0.39 (0.17-0.92) | 0.03 | 58.21% |  | 2.50 (1.39-4.49) | 0.00 | 58.21% |  | 1.74 (0.88-3.46) | 0.11 | 58.21% |
| Pheochromocytoma and Paraganglioma(PCPG) | 4.86 (0.55-42.66) | 0.15 | 96.39% |  | 0.16 (0.02-1.36) | 0.09 | 96.39% |  | 4.67 (0.53-41.11) | 0.16 | 96.39% |
| Prostate adenocarcinoma(PRAD) | 0.33 (0.07-1.50) | 0.15 | 98.46% |  | 0.39 (0.09-1.77) | 0.22 | 98.47% |  | 0.36 (0.07-1.90) | 0.23 | 98.47% |
| Rectum adenocarcinoma(READ) | 1.77 (0.36-8.56) | 0.48 | 89.55% |  | 1.98 (0.44-8.93) | 0.38 | 90.00% |  | 11.92 (1.38-103.24) | 0.02 | 90.00% |
| Sarcoma(SARC) | 1.45 (0.92-2.30) | 0.11 | 68.12% |  | 1.69 (1.03-2.75) | 0.04 | 68.56% |  | 0.45 (0.22-0.90) | 0.02 | 68.56% |
| Skin Cutaneous Melanoma(SKCM) | 12.94 (2.03-82.46) | 0.01 | 71.88% |  | 1.96 (0.38-10.14) | 0.42 | 74.29% |  | 0.07 (0.01-0.76) | 0.03 | 74.29% |
| Stomach adenocarcinoma(STAD) | 1.43 (0.89-2.30) | 0.14 | 71.94% |  |  |  |  |  |  |  |  |
| Testicular Germ Cell Tumors(TGCT) | 3.92 (0.24-62.66) | 0.33 | 98.36% |  | 0.42 (0.03-6.64) | 0.53 | 98.36% |  | 2.22 (0.14-35.52) | 0.57 | 98.36% |
| Thymoma(THYM) | 2.45 (0.48-12.38) | 0.28 | 94.50% |  | 3.60 (0.66-19.67) | 0.14 | 94.29% |  | 0.10 (0.02-0.64) | 0.02 | 94.29% |
| Thyroid carcinoma(THCA) | 5.70 (1.90-17.05) | 0.00 | 96.91% |  | 1.94 (0.67-5.63) | 0.22 | 96.90% |  | 0.25 (0.07-0.91) | 0.04 | 96.90% |
| Uterine Carcinosarcoma(UCS) | 0.35 (0.14-0.87) | 0.02 | 43.64% |  | 2.13 (0.81-5.60) | 0.12 | 43.64% |  | 1.89 (0.90-3.96) | 0.09 | 43.64% |
| Uterine Corpus Endometrial Carcinoma(UCEC) | 1.42 (0.71-2.84) | 0.32 | 90.44% |  | 1.41 (0.54-3.69) | 0.48 | 90.99% |  | 0.43 (0.16-1.12) | 0.08 | 90.99% |
| Uveal Melanoma(UVM) | 0.40 (0.12-1.32) | 0.13 | 79.37% |  | 4.57 (1.00-20.94) | 0.05 | 79.37% |  | 3.01 (0.90-10.09) | 0.07 | 79.37% |

| **Supplementary Table S2b The frequency of Ras mutation in different tumors** | | |
| --- | --- | --- |
| **Cancer Types** | **Frequency of Ras mutantion** | |
|  | **Number of pateints harboring Ras mutation** | **Percentage of patients harboring Ras mutation** |
| Acute Myeloid Leukemia (TCGA, Provisional) | 23 | 11.70% |
| Bladder Urothelial Carcinoma (TCGA, Provisional) | 8 | 6.20% |
| Brain Lower Grade Glioma (TCGA, Provisional) | 2 | 0.70% |
| Breast Invasive Carcinoma (TCGA, Provisional) | 10 | 1% |
| Cervical Squamous Cell Carcinoma and Endocervical Adenocarcinoma (TCGA, Provisional) | 12 | 6.20% |
| Cholangiocarcinoma (TCGA, Provisional) | 2 | 5.70% |
| Colorectal Adenocarcinoma (TCGA, Provisional) | 111 | 49.80% |
| Esophageal Adenocarcinoma (Broad, Nat Genet 2013) | 6 | 4.10% |
| Glioblastoma Multiforme (TCGA, Provisional) | 3 | 1% |
| Head and Neck Squamous Cell Carcinoma (TCGA, Provisional) | 12 | 4.30% |
| Kidney Chromophobe (TCGA, Provisional) | 1 | 1.50% |
| Kidney Renal Clear Cell Carcinoma (TCGA, Provisional) | 2 | 0.50% |
| Kidney Renal Papillary Cell Carcinoma (TCGA, Provisional) | 3 | 1.90% |
| Liver Hepatocellular Carcinoma (TCGA, Provisional) | 5 | 2.50% |
| Lung Adenocarcinoma (TCGA, Provisional) | 77 | 33.50% |
| Lung Squamous Cell Carcinoma (TCGA, Provisional) | 8 | 4.50% |
| Lymphoid Neoplasm Diffuse Large B-cell Lymphoma (TCGA, Provisional) | 3 | 6.30% |
| Medulloblastoma (ICGC, Nature 2012) | 1 | 0.80% |
| **Ovarian Serous Cystadenocarcinoma (TCGA, Provisional)** | **4** | **1.30%** |
| Pancreatic Adenocarcinoma (TCGA, Provisional) | 123 | 84.20% |
| Pheochromocytoma and Paraganglioma (TCGA, Provisional) | 18 | 9.80% |
| Prostate Adenocarcinoma (TCGA, Provisional) | 4 | 1.20% |
| Sarcoma (TCGA, Provisional) | 3 | 1.20% |
| Skin Cutaneous Melanoma (TCGA, Provisional) | 105 | 30.40% |
| Stomach Adenocarcinoma (TCGA, Provisional) | 31 | 10.70% |
| Testicular Germ Cell Cancer (TCGA, Provisional) | 28 | 18.10% |
| Thyroid Carcinoma (TCGA, Provisional) | 52 | 12.80% |
| Uterine Carcinosarcoma (TCGA, Provisional) | 8 | 14% |
| Uterine Corpus Endometrial Carcinoma (TCGA, Provisional) | 60 | 24.20% |

| **Supplementary Table S2c Correlation of Fra-1 and miR-134 in different tumor tissues** | | | |
| --- | --- | --- | --- |
| **Cancer Types** | **Tumor samples** | | |
|  | **N** | **R** | **P-value** |
| Kidney Chromophobe(KICH) | 66 | 0.52 | 0.00 |
| Mesothelioma(MESO) | 87 | 0.33 | 0.00 |
| Thyroid carcinoma(THCA) | 504 | 0.30 | 0.00 |
| Uveal Melanoma(UVM) | 80 | 0.30 | 0.01 |
| Lymphoid Neoplasm Diffuse Large B-cell Lymphoma(DLBC) | 47 | 0.27 | 0.07 |
| Kidney renal clear cell carcinoma(KIRC) | 515 | 0.27 | 0.00 |
| Kidney renal papillary cell carcinoma(KIRP) | 290 | 0.26 | 0.00 |
| Rectum adenocarcinoma(READ) | 90 | 0.25 | 0.02 |
| Adrenocortical carcinoma(ACC) | 79 | 0.25 | 0.03 |
| Sarcoma(SARC) | 257 | 0.23 | 0.00 |
| Lung adenocarcinoma(LUAD) | 447 | 0.21 | 0.00 |
| Lung squamous cell carcinoma(LUSC) | 342 | 0.20 | 0.00 |
| **Ovarian serous cystadenocarcinoma(OV)** | **408** | **0.20** | **0.00** |
| Prostate adenocarcinoma(PRAD) | 493 | 0.20 | 0.00 |
| Liver hepatocellular carcinoma(LIHC) | 367 | 0.20 | 0.00 |
| Skin Cutaneous Melanoma(SKCM) | 449 | 0.17 | 0.00 |
| Colon adenocarcinoma(COAD) | 235 | 0.16 | 0.01 |
| Bladder urothelial carcinoma(BLCA) | 405 | 0.15 | 0.00 |
| Cervical and endocervical cancers(CESC) | 304 | 0.13 | 0.02 |
| Cholangiocarcinoma(CHOL) | 36 | 0.13 | 0.47 |
| Brain Lower Grade Glioma(LGG) | 512 | 0.12 | 0.01 |
| Breast invasive carcinoma(BRCA) | 753 | 0.10 | 0.01 |
| Pheochromocytoma and Paraganglioma(PCPG) | 179 | 0.09 | 0.22 |
| Uterine Corpus Endometrial Carcinoma(UCEC) | 238 | 0.09 | 0.18 |
| Head and Neck squamous cell carcinoma(HNSC) | 478 | 0.06 | 0.16 |
| Acute Myeloid Leukemia(LAML) | 173 | 0.05 | 0.52 |
| Testicular Germ Cell Tumors(TGCT) | 150 | 0.04 | 0.62 |
| Thymoma(THYM) | 120 | -0.04 | 0.63 |
| Uterine Carcinosarcoma(UCS) | 57 | -0.18 | 0.19 |
| Pancreatic adenocarcinoma(PAAD) | 178 | -0.20 | 0.01 |

| **Supplemental Table S3 Oligonucleotides used for real-time RT-PCR, qChIP, plasmid construction and siRNA** | | |
| --- | --- | --- |
| **Gene/Fragment** | **Forward** | **Reverse** |
| **Oligonucleotides used for real-time RT-PCR** | |  |
| c-Fos | GCCTCTCTTACTACCACTCACC | AGATGGCAGTGACCGTGGGAAT |
| Fos-B | TCTGTCTTCGGTGGACTCCTTC | GTTGCACAAGCCACTGGAGGTC |
| Fra-1 | CAGCTCATCGCAAGAGTAGCA | CAAAGCGAGGAGGGTTGGA |
| Fra-2 | AAGAGGAGGAGAAGCGTCGCAT | GCTCAGCAATCTCCTTCTGCAG |
| c-Jun | CCTTGAAAGCTCAGAACTCGGAG | TGCTGCGTTAGCATGAGTTGGC |
| JunB | CGATCTGCACAAGATGAACCACG | CTGCTGAGGTTGGTGTAAACGG |
| JunD | ATCGACATGGACACGCAGGAGC | CTCCGTGTTCTGACTCTTGAGG |
| Pri-miR-134 | AACCCTCAGCATCACTCCCACT | TCATCGCAGTCCACCAAGCA |
| TCF21 | CACTTGAGGCAGATCCTGGCTA | CGGTCACCACTTCTTTCAGGTC |
| SDS22 | TCACCAACCTGACAGTCCTCAG | CGATGCCATTGTGGCTAAGGTAC |
| PPP1R12A | GCAGGTGTTACACGTTCAGCTTC | GATGTACTGGCTAGTCGTCTTGG |
| GAPDH | ACCCACTCCTCCACCTTTG | CACCACCCTGTTGCTGTAG |
| Uncut | GCACCAAAATCAACGGGACT | AAAGCACGAGATTCTTCGCC |
| Joint | GCACCAAAATCAACGGGACT | GCTGAACTTGTGGCCGTTTA |
| RPS20 promoter | AAGTTCTTTCTTTTTGAGGAAGACG | GAACAGCGGTGAGTCAGGA |
| **Oligonucleotides used for qChIP** | |  |
| miR134P-F1 | CTCCGTGGCTTTGTCCTATCTG | CTTCTCCTGAGGGTGGAATGAA |
| miR134P-F2 | TCAGCATCCAGGCTACCTACAA | ATGGGTCATCCTTACGAGTATTTCA |
| miR134P-F3 | TGGCTCGGTAGTGTTACTGTCATC | TGCTTCCAGAAACCCTTCCCT |
| miR134P-F4 | CTAGGTGATTCTGGACCTTAGCA | ACTGTAACCCGCTTTCCCTTT |
| miR134P-F5 | CTGGCACCTTGATGTGCTTGA | TGGAGTTGAAATTGTGGTCCTG |
| miR134P-F6 | ATGCTCGGTGTCCACTCTGTCC | CTATCTTTCCTGAAGCTGCCATT |
| ChIP for γ-H2AX | CCAAGTCTCCACCCCATTGA | GCGGATCTGACGGTTCACTA |
| **Oligonucleotides used for plasmid construction** | |  |
| miR134P-P1 | ctaGCTAGCTGGGAGTCGCTGGGTCTGAG | ggaAGATCTTCTGGAAGTCGGCCTGGG |
| miR134P-P2 | ctaGCTAGCCAAGGTATGTATGTGAACGG | ggaAGATCTTCTGGAAGTCGGCCTGGG |
| miR134P-P3 | ctaGCTAGCTCCGTGGCTTTGTCCTATCTG | ggaAGATCTTCTGGAAGTCGGCCTGGG |
| TCF21-3'UTR | CTAGTCTAGATTGGAGGTGCGAGTCTGG | CTAGTCTAGACAAAAGTAGCAAAACTTGTTTG |
| SDS22-3'UTR | CTAGTCTAGATTCTTGGCTCCTCATGTGGTC | CTAGTCTAGACAGTGTGGCAAGATTTAATTG |
| PPP1R12-3'UTR | CTAGTCTAGAATGGAATTGCACATATTAGTAAC | CTAGTCTAGACTGCCACATGAAGAGTTTAGAAG |
| SDS22-mut-3'UTR | GTCGTCACTATATCAAGACTGTGAAACCCAATGGC | GCCATTGGGTTTCACAGTCTTGATATAGTGACGAC |
| **Oligonucleotides used for siRNA** | |  |
| siFRA-1 | CACCAUGAGUGGCAGUCAGdTdT | CUGACUGCCACUCAUGGUGdTdT |
| siSDS22 | GCCACAAUGGCAUCGAGGUdTdT | ACCUCGAUGCCAUUGUGGCdTdT |
| siHRas^v12^ | CGCCGUCGGUGUGGGCAAGdTdT | CUUGCCCACACCGACGGCGdTdT |
