# Supplementary material for: The Fra-1–miR-134–SDS22 feedback loop amplifies ERK/JNK signaling and reduces chemosensitivity in ovarian cancer cells
Source: Cell Death Dis. 2016 Sep 29;7(9):e2384–. doi: 10.1038/cddis.2016.289 (PMC5059884; doi:10.1038/cddis.2016.289)
Supplement: Supplementary Figure Legends [file cddis2016289x2.doc]

**Supplementary Data**

**Supplementary Figure Legends**

**Supplementary Figure S1. Oncogenic H-Ras induces miR-134 expression. (a)** H-Ras protein levels (left) and relative miR-134 expression levels (right) in T29 cells transiently transfected with pCMV-H-Ras^V12^ (pH-Ras^V12^) or pcDNA3.1. **(b)** H-Ras protein levels (left) and relative miR-134 expression levels (right) in T29H cells transfected with H-Ras^V12^ siRNA (siHRas^V12^) or control siRNA (siCONT). **(c)** Relative miR-134 expression levels in T29H cells treated with 10 μM FTI-277 for 24 h. Data are shown as mean ± SD from three independent experiments. ***P*<0.01 by Student’s *t*-test.

**Supplementary Figure S2.** The representative Western blot of Fra-1 after chromatin immunoprecipitation (ChIP) assays with anti-Fra-1 in T29H and T29 cells.

**Supplementary Figure S3. Identification of miR-134 target genes. (a)** Venn diagram of the candidate target genes predicated by 3 independent algorithms. **(b)** The putative miR-134-binding sites in the SDS22, TCF21 and PPP1R12A 3'-UTRs. The alignment between the putative miR-134 target sites and miR-134 is denoted in gray. The primers used to amplify the 3'-UTR fragment are indicated by red arrows, and the sequences of these primers are listed in Supplementary Table S3. **(c)** A schematic of the reporter constructs. The 3'-UTRs of SDS22, TCF21 and PPP1R12A were inserted into the pGL3-control vector at the Xba1 site immediately downstream of the stop codon of the sequence encoding firefly luciferase. SV40, SV40 promoter; Luc, luciferase coding sequence; 3'-UTR, fragment of the corresponding 3'-UTR; poly A, poly(A) tail. **(d)** HEK293T and T29 cells were co-transfected with miR-134, a luciferase plasmid (pGL3-control, Luc-SDS22 3’UTR, Luc-TCF21 3’UTR or Luc-PPP1R12A 3’UTR) and a pRL-CMV reporter plasmid. The luciferase activity was measured 24 h after transfection. The *y*-axis represents the relative luciferase activity. **(e)** Relative SDS22 mRNA expression levels in miR-134- or In-miR-134-transfected cells. Data are shown as mean ± SD from three independent experiments. **P*<0.05 by Student’s *t*-test.

**Supplementary Figure S4.** The relative expression levels of miR-134 in ES2 cells treated with adriamycin (0.5 μM) **(a)** and etoposide (5 μM) **(b)** for 24 h. Data are shown as mean ± SD from three independent experiments. **P*<0.05, ***P*<0.01 by Student’s *t*-test.

**Supplementary Figure S5.** Left, representative comet assay showing formation of DNA breaks (formation of a “comet tail”) in miR-134-transfected cells after 12 h of etoposide (5 μM) treatment and a 12-h recovery. Right, box plot graph showing Tail DNA%. Data are shown as mean ± SD from three independent experiments. **P*<0.05, ***P*<0.01 by Student’s *t*-test.

**Supplementary Figure S6. NHEJ assays system for detecting chromosomal DSBs *in vivo*. (a)** Schematic of the NHEJ assays system. Two I-SceI sites in the reverse orientation are indicated by yellow arrow heads. Locations of the quantitative PCR primers used to evaluate I-SceI-induced DSB (uncut DNA) and the subsequent joining product (joined DNA) are indicated by blue and green arrows, respectively. The ChIP primers are indicated by red arrows. CMV, cytomegalovirus promoter; HPH, hygromycin B phosphotransferase. **(b-d)** Assessment of DSB generation and DNA end-joining in SKOV3-Hygro-EGFP cells. **(b)** The proportion of uncut DNA was assessed using quantitative PCR and expressed as a ratio of the amount of uncut DNA before the pCBA-I-SceI plasmid transfection. **(c)** The proportion of joined DNA was assessed by quantitative PCR and expressed as a ratio of the amount of joined DNA 48 h after transfection of the pCBA-I-SceI plasmid. **(d)** The proportion of EGFP-positive cells was assessed by fluorescence-activated cell sorting analysis. Data are shown as mean ± SD from three independent experiments.

**Supplementary Figure S7. miR-134 promotes cell proliferation, migration and invasion in T29 cells. (a and b)** T29 cells were transfected with siSDS22 or siCONT. **(a)** The cell proliferation assay was performed at the indicated time points. **(b)** Representative micrographs of cell migration and invasion assay (left), and the quantification (right). **(c and d)** T29 cells were transfected with miR-134 or the control mimics. **(c)** The cell proliferation assay was performed at the indicated time points. **(d)** Representative micrographs of cell migration and invasion assays (left), and the quantification (right). **(e-g)** T29 cells were co-transfected with miR-con/miR-134 and pcDNA3.1/pSDS22 as indicated. **(e)** SDS22 expression detected by Western blot. **(f)** Cell proliferation was measured 96 h after transfection. **(g)** Representative micrographs of cell migration and invasion assays (left), and the quantification (right). Data are shown as mean ± SD from three independent experiments. **P*<0.05, ***P*<0.01 by Student’s *t*-test.

**Supplementary Figure S8.** The levels of SDS22, p-JNK, p-ERK and Fra-1 in 6 paired subcutaneous xenografts of T29H cells transfected with the miR-134 antagomir (I) and the control antagomir (C). The number represents the paired tumors from same nude mice.

**Supplementary Figure S9.** The levels of miR-134 (top) and the protein levels of SDS22 (bottom) in human primary ovarian cancer specimens. The number represents the Patients No.
