# Supplementary material for: The Fra-1–miR-134–SDS22 feedback loop amplifies ERK/JNK signaling and reduces chemosensitivity in ovarian cancer cells
Source: Cell Death Dis. 2016 Sep 29;7(9):e2384–. doi: 10.1038/cddis.2016.289 (PMC5059884; doi:10.1038/cddis.2016.289)
Supplement: Supplementary Figures [file cddis2016289x4.pdf]

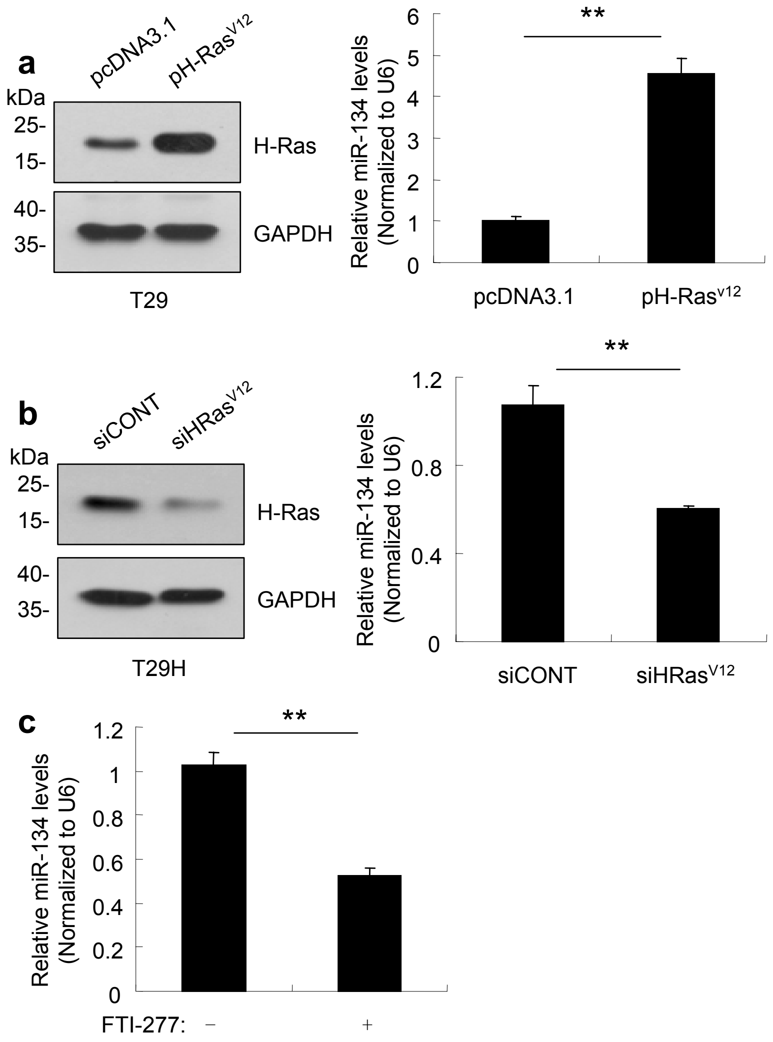

Supplementary Fig. S1

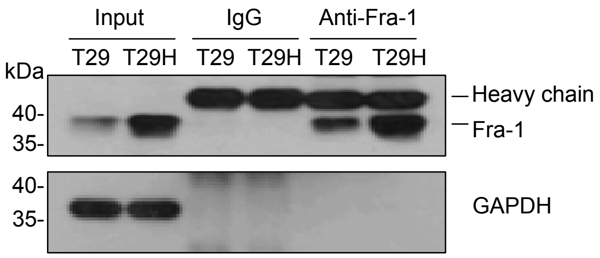

Supplementary Fig. S2

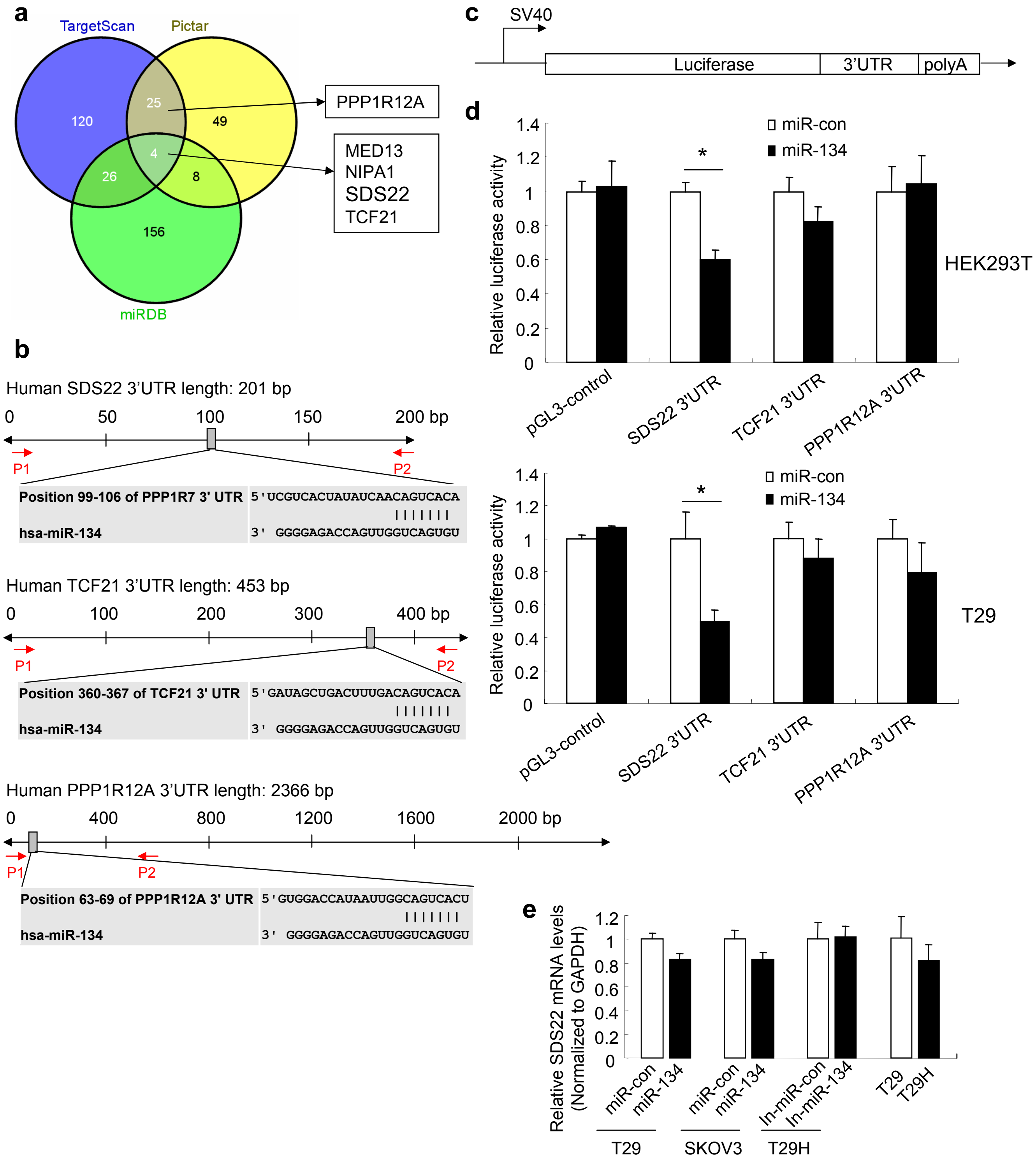

Supplementary Fig. S3

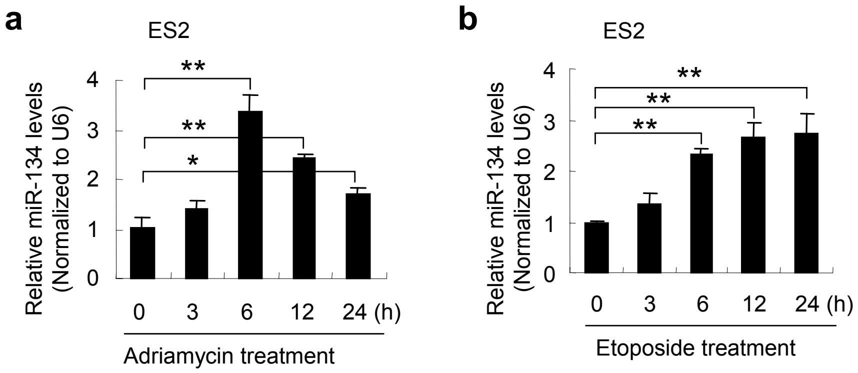

Supplementary Fig. S4

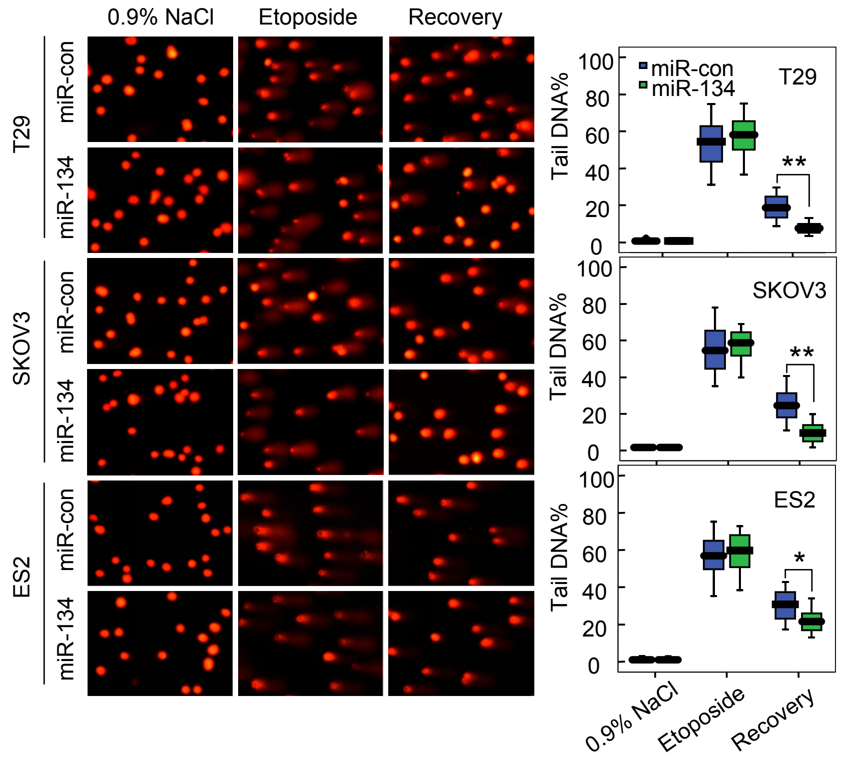

Supplementary Fig. S5

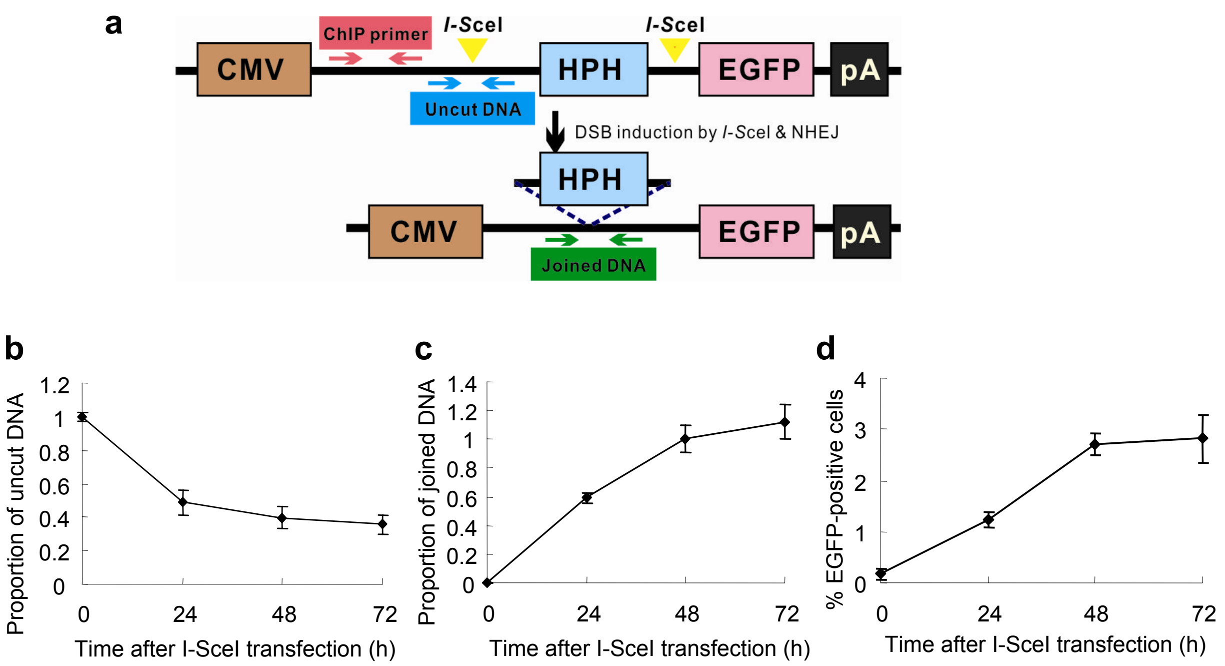

Supplementary Fig. S6

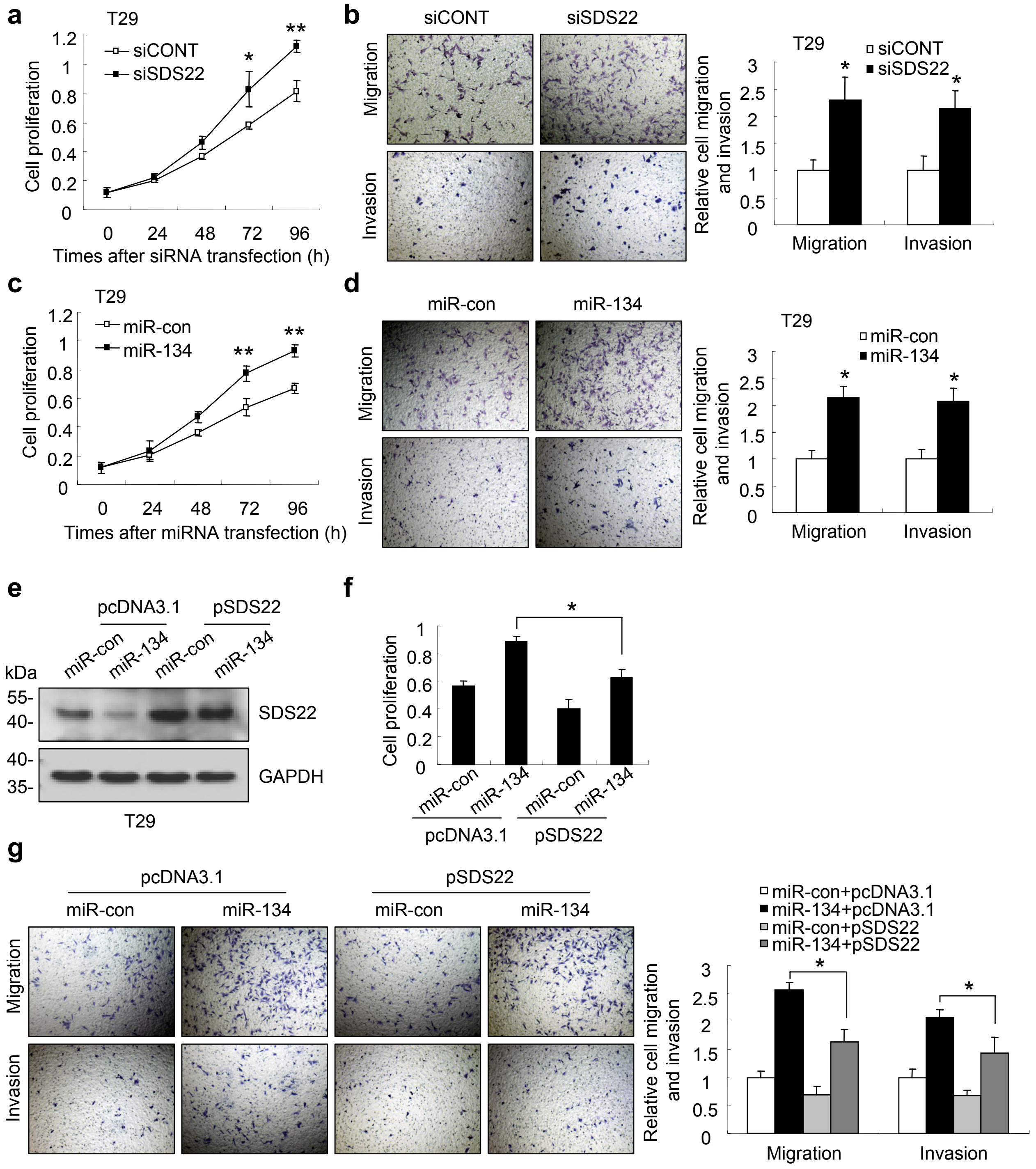

Supplementary Fig. S7

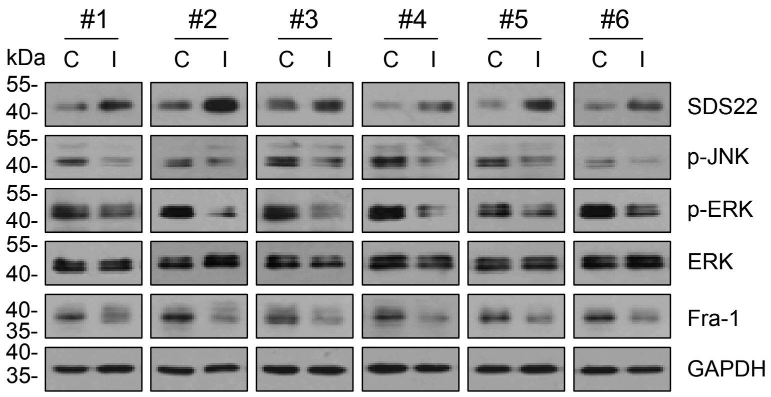

Supplementary Fig. S8

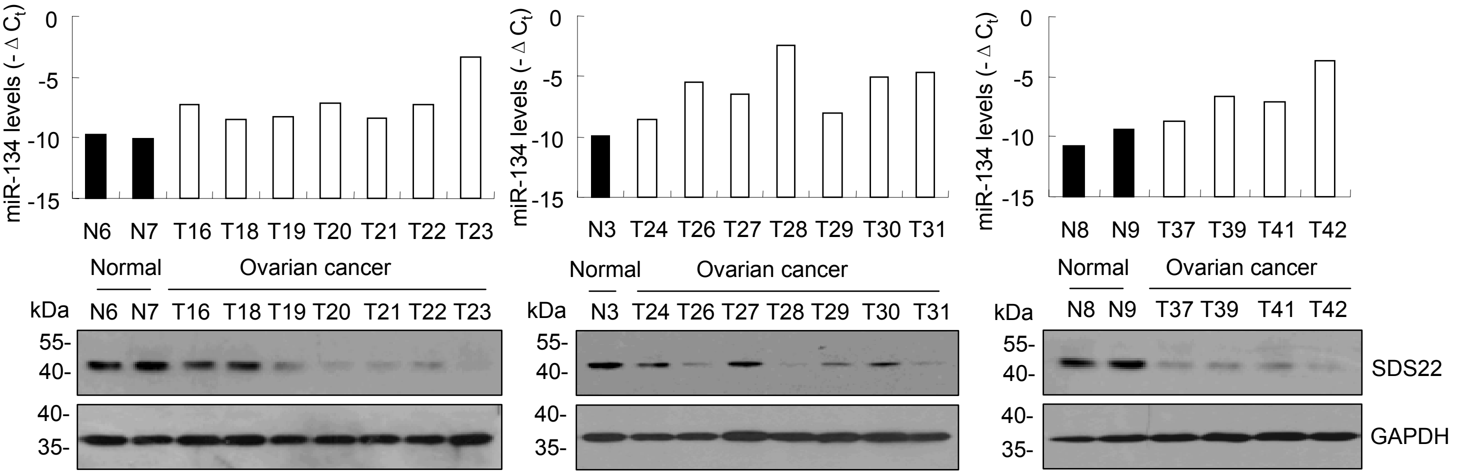

Supplementary Fig. S9
